# Supplementary material for: CINner: Modeling and simulation of chromosomal instability in cancer at single-cell resolution
Source: PLoS Comput Biol. 2025 Apr 3;21(4):e1012902. doi: 10.1371/journal.pcbi.1012902 (PMC11990800; doi:10.1371/journal.pcbi.1012902)
Supplement: S1 Table — Estimation of chromosome-arm selection parameters (Figs 2 and S1–S17) was performed with non-WGD samples. Estimation of WGD probability and WGD-associated CIN (Figs 4, S22 and S23) employed WGD samples. *: Cancer types with WGD proportion ≤0.1 were excluded from the WGD inference. (DOCX) [file pcbi.1012902.s002.docx]

| PCAWG cancer type | Number of samples | Number of non-WGD samples | Number of WGD samples | WGD proportion |
| --- | --- | --- | --- | --- |
| Breast-AdenoCA | 81 | 34 | 47 | 0.58 |
| ColoRect-AdenoCA | 58 | 37 | 21 | 0.36 |
| Prost-AdenoCA | 19 | 19 | 0 | 0* |
| CNS-GBM | 41 | 34 | 7 | 0.17 |
| Head-SCC | 43 | 24 | 19 | 0.44 |
| Kidney-RCC | 68 | 60 | 8 | 0.11 |
| Stomach-AdenoCA | 35 | 18 | 17 | 0.48 |
| Liver-HCC | 49 | 31 | 18 | 0.36 |
| Thy-AdenoCA | 47 | 47 | 0 | 0* |
| Kidney-ChRCC | 42 | 33 | 9 | 0.21 |
| Skin-Melanoma | 35 | 16 | 19 | 0.54 |
| CNS-Oligo | 17 | 16 | 1 | 0.05* |
| Lung-AdenoCA | 34 | 14 | 20 | 0.58 |
| Lung-SCC | 43 | 14 | 29 | 0.67 |
| Ovary-AdenoCA | 41 | 20 | 21 | 0.51 |
| Uterus-AdenoCA | 49 | 32 | 17 | 0.34 |
| Cervix-SCC | 16 | 10 | 6 | 0.37 |
