# Supplementary material for: CINner: Modeling and simulation of chromosomal instability in cancer at single-cell resolution
Source: PLoS Comput Biol. 2025 Apr 3;21(4):e1012902. doi: 10.1371/journal.pcbi.1012902 (PMC11990800; doi:10.1371/journal.pcbi.1012902)
Supplement: S2 Table — Green indicates properties of cell-specific fitness, CNA mechanisms or aspects of the mutational process that an algorithm incorporates. Red indicates the algorithm does not include such properties. Yellow indicates properties that are included, with important caveats. †: CN breakpoint (loci where CN changes between genomic regions) can be directly computed from simulated CN profiles. *: cell fitness is modeled based on arm-specific selection coefficients computed from pan-cancer data [22]. ‡: CN profiles are input from observed data and not simulated. A: inference for mouse T-cell lymphoma and human colon cancer organoid single-cell data. B: inference for breast cancer. C: inference for yeast. (DOCX) [file pcbi.1012902.s003.docx]

| Algorithms | Fitness inference | Copy Number Aberrations | | | | | | | | Mutations | |
| --- | --- | --- | --- | --- | --- | --- | --- | --- | --- | --- | --- |
|  |  | CN signature | Chromosomal rearrangement | WGD | Whole-chromosome missegregation | Chromosome-arm missegregation | Focal amplification/deletion | Allele-specific CNA | CN breakpoints | (Selective) driver mutations | (Neutral) passenger mutations |
| CINner |  |  |  |  |  |  |  |  | $\dagger$ |  |  |
| CINsim [21] | A |  |  |  |  |  |  |  | $\dagger$ |  |  |
| [62] | B |  |  |  |  |  |  |  |  |  |  |
| [13] and [28] | C |  |  |  |  |  |  |  | $\dagger$ |  |  |
| [18] | * |  |  |  |  |  |  |  | $\dagger$ |  |  |
| [68] | * |  |  |  |  |  |  |  | $\dagger$ |  |  |
| [19] | * |  |  |  |  |  |  |  | $\dagger$ |  |  |
| CINSignatureGenomeSimulation [63] |  |  |  |  |  |  |  |  | $\dagger$ |  |  |
| [66] |  |  |  |  |  |  |  |  | $\dagger$ |  |  |
| SimClone [20] |  |  | $\ddagger$ | $\ddagger$ | $\ddagger$ | $\ddagger$ | $\ddagger$ |  | $\dagger$ |  |  |
| [60] and [61] |  |  |  |  |  |  |  |  | $\dagger$ |  |  |
| [67] |  |  |  |  |  |  |  |  | $\dagger$ |  |  |
| CellCoal [65] |  |  |  |  |  |  |  |  |  |  |  |
