# Supplementary figures and images for: CINner: Modeling and simulation of chromosomal instability in cancer at single-cell resolution
Source: PLoS Comput Biol. 2025 Apr 3;21(4):e1012902. doi: 10.1371/journal.pcbi.1012902 (PMC11990800; doi:10.1371/journal.pcbi.1012902)

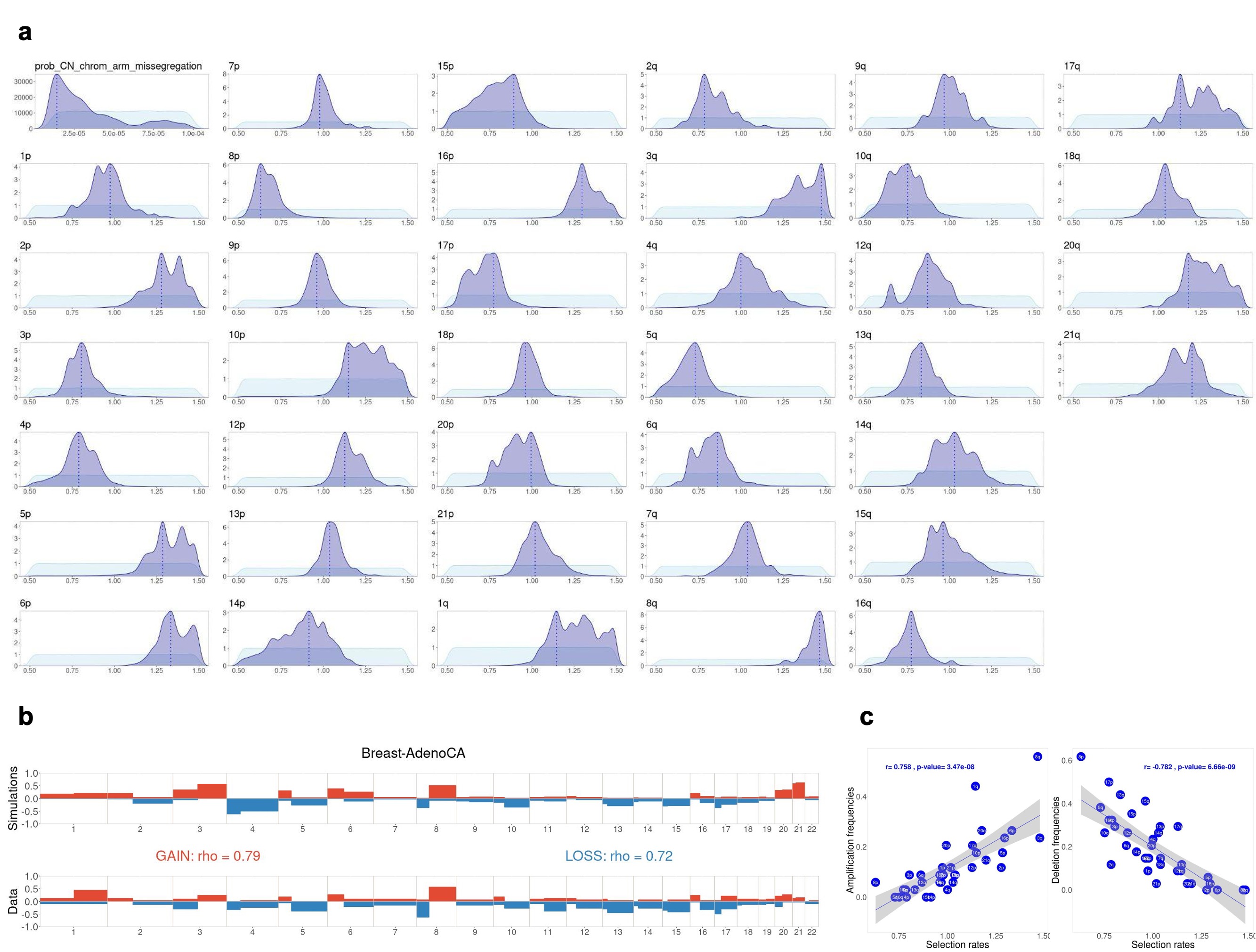

Supplement: S1 Fig — (a) Prior distribution (light blue) and posterior distribution (dark blue) from inference with ABC random forest. Broken line represents the mode in the posterior distribution for each parameter. (b) Comparison between simulations with fitted parameter (top) and gain/loss frequencies at arm level from TCGA (bottom). The simulations are computed with the posterior modes from (a). Spearman’s correlation coefficient rho between frequencies of gains (or losses) among each arm in PCAWG and simulations. (c) Correlation between inferred selection rates and amplification/deletion frequencies for individual chromosome arms. Linear regressions and p-values from Pearson correlation. (JPG) [file pcbi.1012902.s004.jpg]

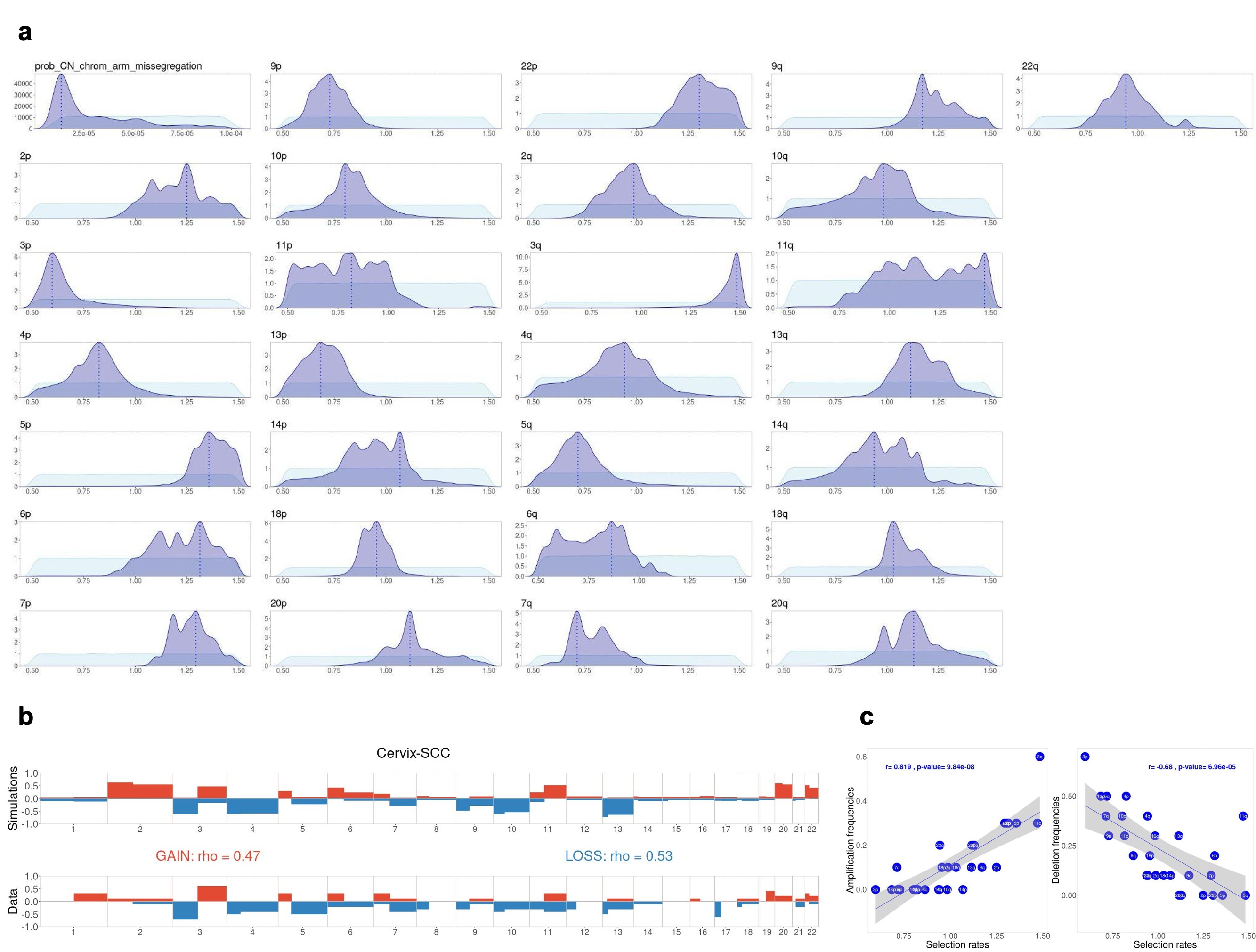

Supplement: S2 Fig — (a) Prior distribution (light blue) and posterior distribution (dark blue) from inference with ABC random forest. Broken line represents the mode in the posterior distribution for each parameter. (b) Comparison between simulations with fitted parameter (top) and gain/loss frequencies at arm level from TCGA (bottom). The simulations are computed with the posterior modes from (a). Spearman’s correlation coefficient rho between frequencies of gains (or losses) among each arm in PCAWG and simulations. (c) Correlation between inferred selection rates and amplification/deletion frequencies for individual chromosome arms. Linear regressions and p-values from Pearson correlation. (JPG) [file pcbi.1012902.s005.jpg]

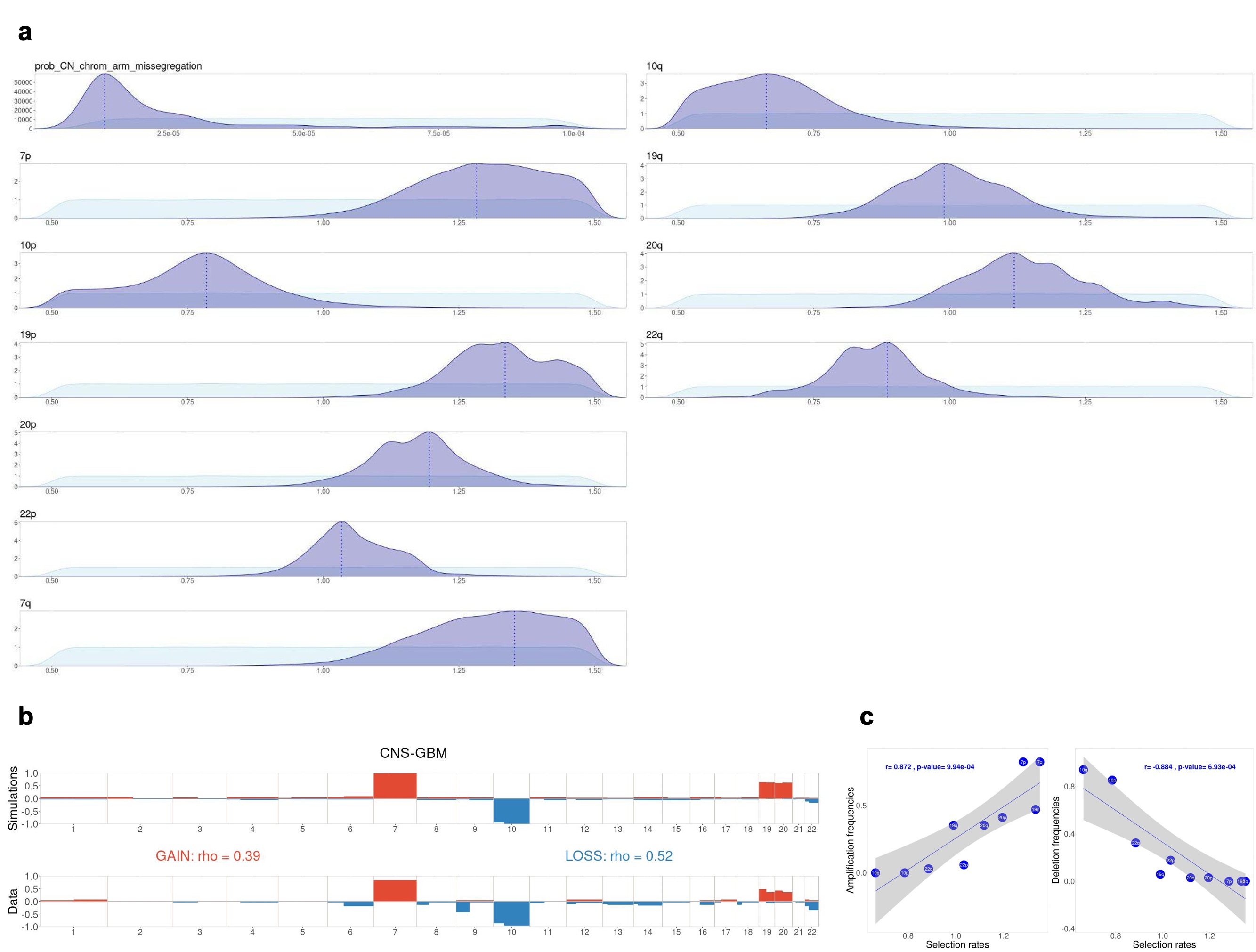

Supplement: S3 Fig — (a) Prior distribution (light blue) and posterior distribution (dark blue) from inference with ABC random forest. Broken line represents the mode in the posterior distribution for each parameter. (b) Comparison between simulations with fitted parameter (top) and gain/loss frequencies at arm level from TCGA (bottom). The simulations are computed with the posterior modes from (a). Spearman’s correlation coefficient rho between frequencies of gains (or losses) among each arm in PCAWG and simulations. (c) Correlation between inferred selection rates and amplification/deletion frequencies for individual chromosome arms. Linear regressions and p-values from Pearson correlation. (JPG) [file pcbi.1012902.s006.jpg]

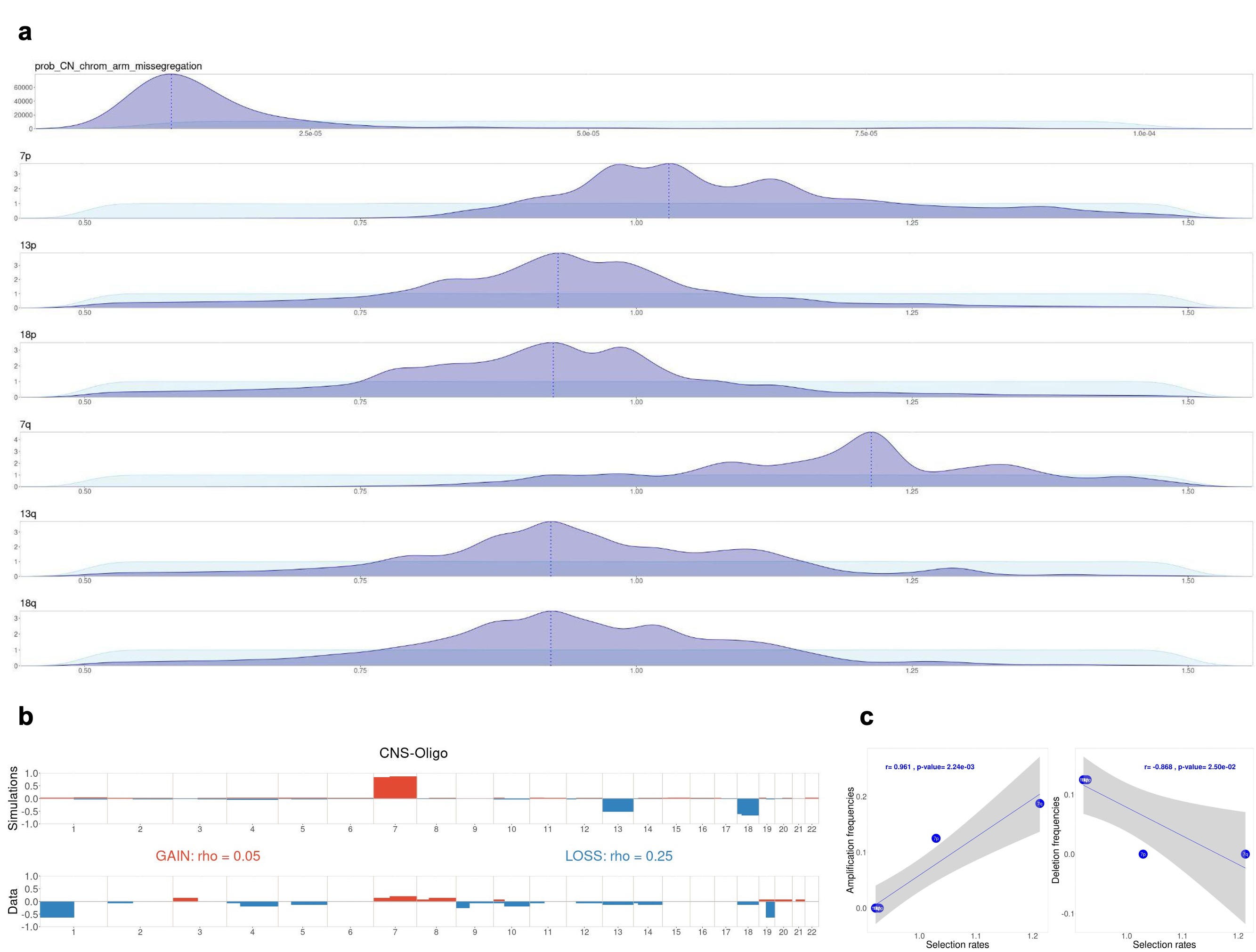

Supplement: S4 Fig — (a) Prior distribution (light blue) and posterior distribution (dark blue) from inference with ABC random forest. Broken line represents the mode in the posterior distribution for each parameter. (b) Comparison between simulations with fitted parameter (top) and gain/loss frequencies at arm level from TCGA (bottom). The simulations are computed with the posterior modes from (a). Spearman’s correlation coefficient rho between frequencies of gains (or losses) among each arm in PCAWG and simulations. (c) Correlation between inferred selection rates and amplification/deletion frequencies for individual chromosome arms. Linear regressions and p-values from Pearson correlation. (JPG) [file pcbi.1012902.s007.jpg]

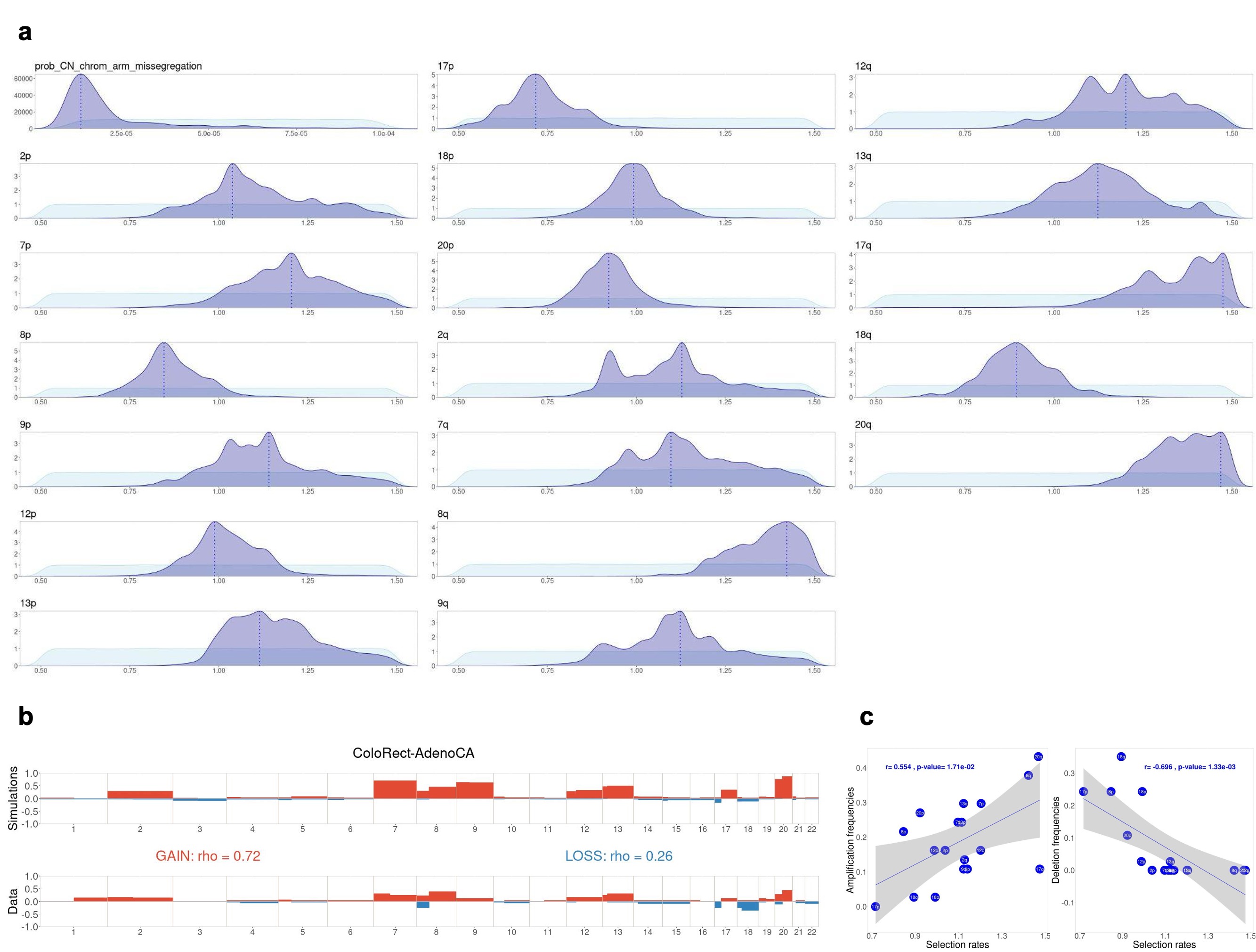

Supplement: S5 Fig — (a) Prior distribution (light blue) and posterior distribution (dark blue) from inference with ABC random forest. Broken line represents the mode in the posterior distribution for each parameter. (b) Comparison between simulations with fitted parameter (top) and gain/loss frequencies at arm level from TCGA (bottom). The simulations are computed with the posterior modes from (a). Spearman’s correlation coefficient rho between frequencies of gains (or losses) among each arm in PCAWG and simulations. (c) Correlation between inferred selection rates and amplification/deletion frequencies for individual chromosome arms. Linear regressions and p-values from Pearson correlation. (JPG) [file pcbi.1012902.s008.jpg]

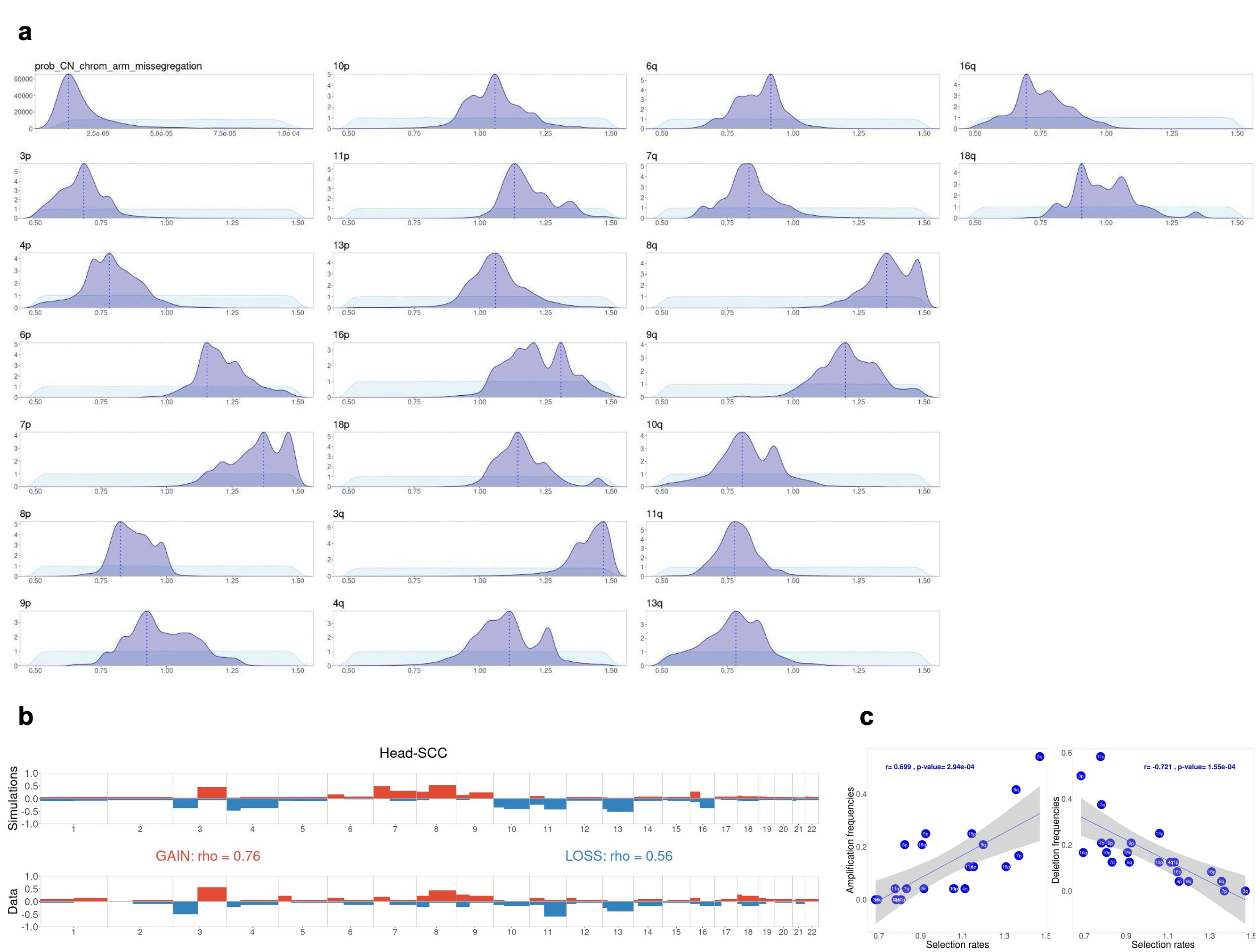

Supplement: S6 Fig — (a) Prior distribution (light blue) and posterior distribution (dark blue) from inference with ABC random forest. Broken line represents the mode in the posterior distribution for each parameter. (b) Comparison between simulations with fitted parameter (top) and gain/loss frequencies at arm level from TCGA (bottom). The simulations are computed with the posterior modes from (a). Spearman’s correlation coefficient rho between frequencies of gains (or losses) among each arm in PCAWG and simulations. (c) Correlation between inferred selection rates and amplification/deletion frequencies for individual chromosome arms. Linear regressions and p-values from Pearson correlation. (JPG) [file pcbi.1012902.s009.jpg]

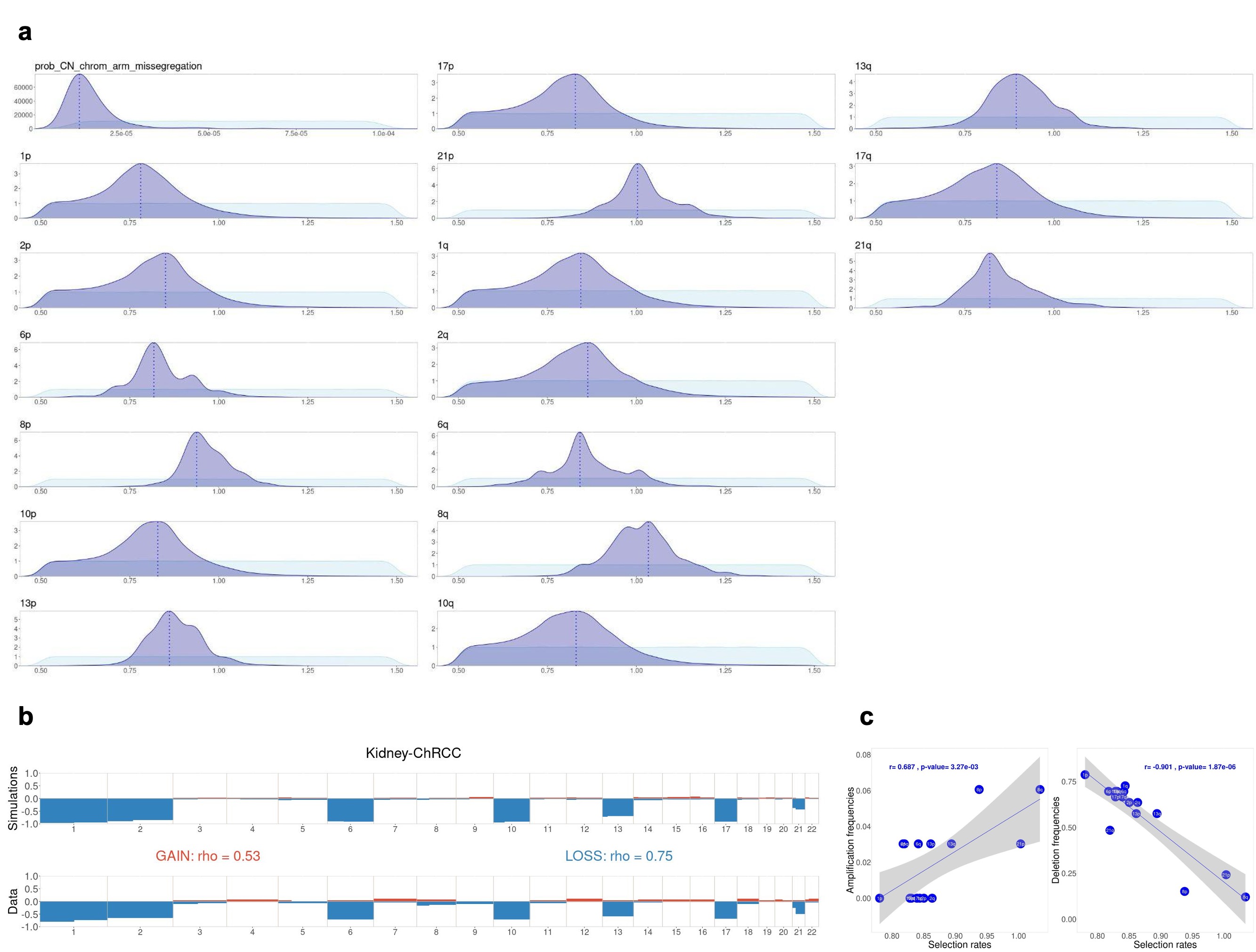

Supplement: S7 Fig — (a) Prior distribution (light blue) and posterior distribution (dark blue) from inference with ABC random forest. Broken line represents the mode in the posterior distribution for each parameter. (b) Comparison between simulations with fitted parameter (top) and gain/loss frequencies at arm level from TCGA (bottom). The simulations are computed with the posterior modes from (a). Spearman’s correlation coefficient rho between frequencies of gains (or losses) among each arm in PCAWG and simulations. (c) Correlation between inferred selection rates and amplification/deletion frequencies for individual chromosome arms. Linear regressions and p-values from Pearson correlation. (JPG) [file pcbi.1012902.s010.jpg]

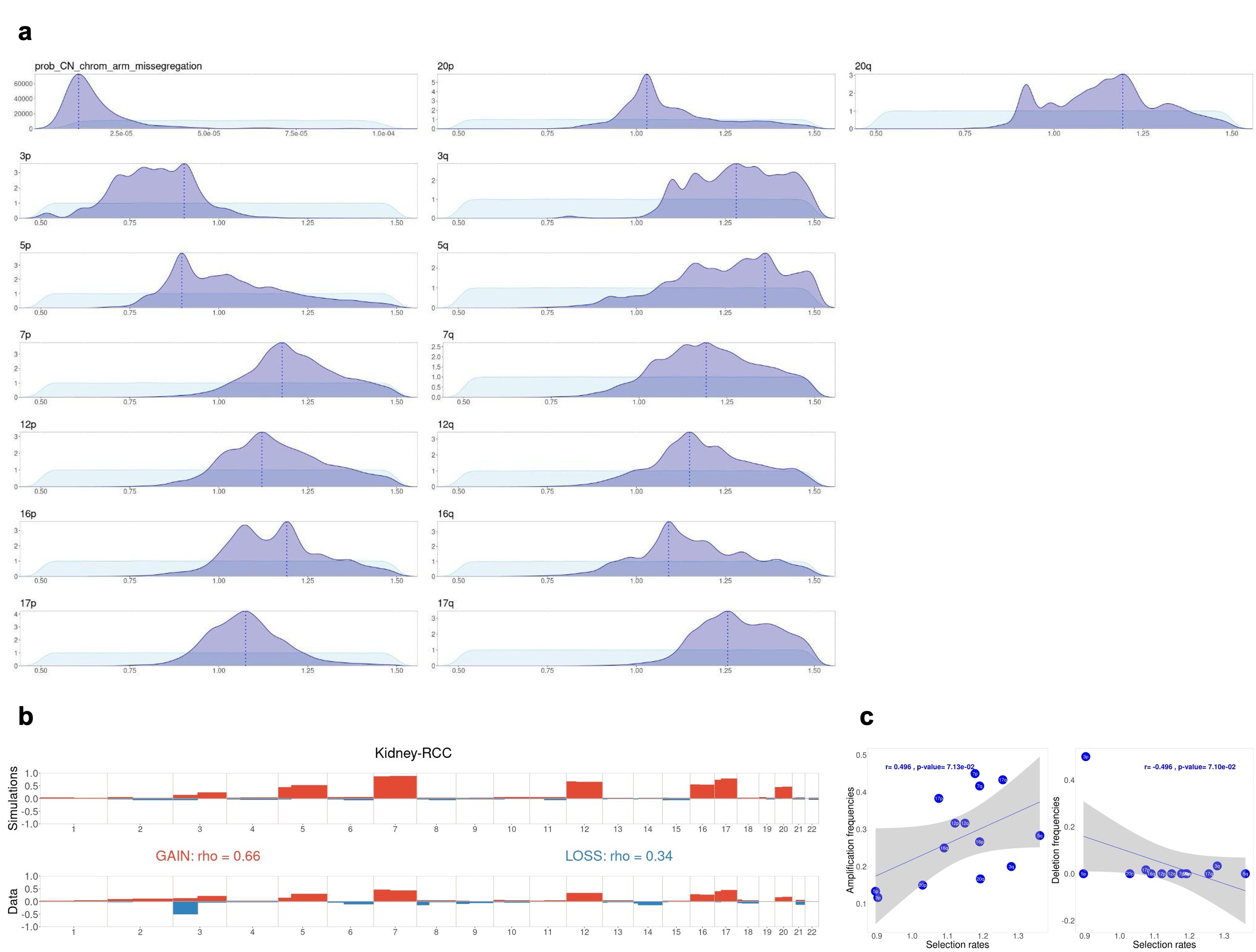

Supplement: S8 Fig — (a) Prior distribution (light blue) and posterior distribution (dark blue) from inference with ABC random forest. Broken line represents the mode in the posterior distribution for each parameter. (b) Comparison between simulations with fitted parameter (top) and gain/loss frequencies at arm level from TCGA (bottom). The simulations are computed with the posterior modes from (a). Spearman’s correlation coefficient rho between frequencies of gains (or losses) among each arm in PCAWG and simulations. (c) Correlation between inferred selection rates and amplification/deletion frequencies for individual chromosome arms. Linear regressions and p-values from Pearson correlation. (JPG) [file pcbi.1012902.s011.jpg]

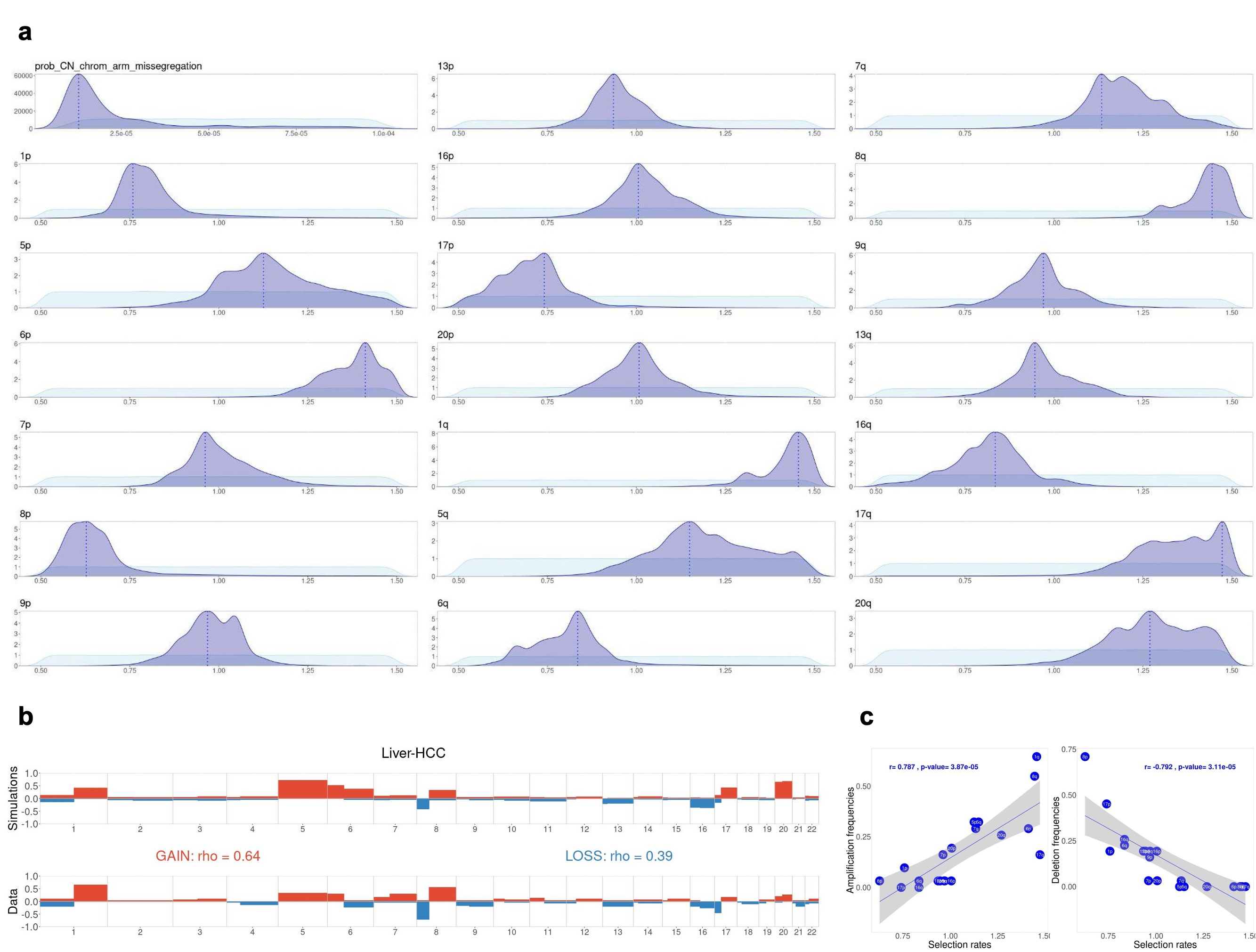

Supplement: S9 Fig — (a) Prior distribution (light blue) and posterior distribution (dark blue) from inference with ABC random forest. Broken line represents the mode in the posterior distribution for each parameter. (b) Comparison between simulations with fitted parameter (top) and gain/loss frequencies at arm level from TCGA (bottom). The simulations are computed with the posterior modes from (a). Spearman’s correlation coefficient rho between frequencies of gains (or losses) among each arm in PCAWG and simulations. (c) Correlation between inferred selection rates and amplification/deletion frequencies for individual chromosome arms. Linear regressions and p-values from Pearson correlation. (JPG) [file pcbi.1012902.s012.jpg]

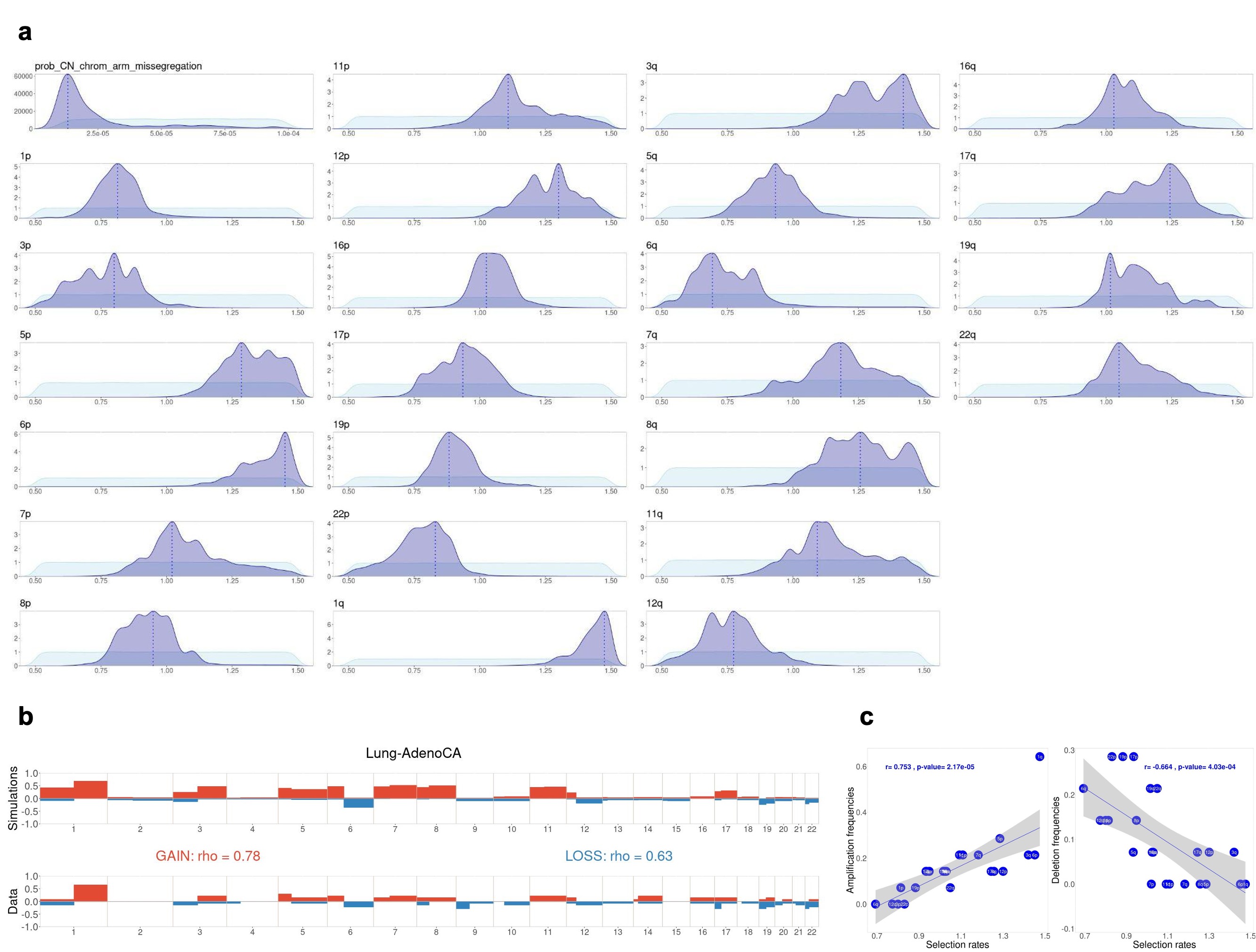

Supplement: S10 Fig — (a) Prior distribution (light blue) and posterior distribution (dark blue) from inference with ABC random forest. Broken line represents the mode in the posterior distribution for each parameter. (b) Comparison between simulations with fitted parameter (top) and gain/loss frequencies at arm level from TCGA (bottom). The simulations are computed with the posterior modes from (a). Spearman’s correlation coefficient rho between frequencies of gains (or losses) among each arm in PCAWG and simulations. (c) Correlation between inferred selection rates and amplification/deletion frequencies for individual chromosome arms. Linear regressions and p-values from Pearson correlation. (JPG) [file pcbi.1012902.s013.jpg]

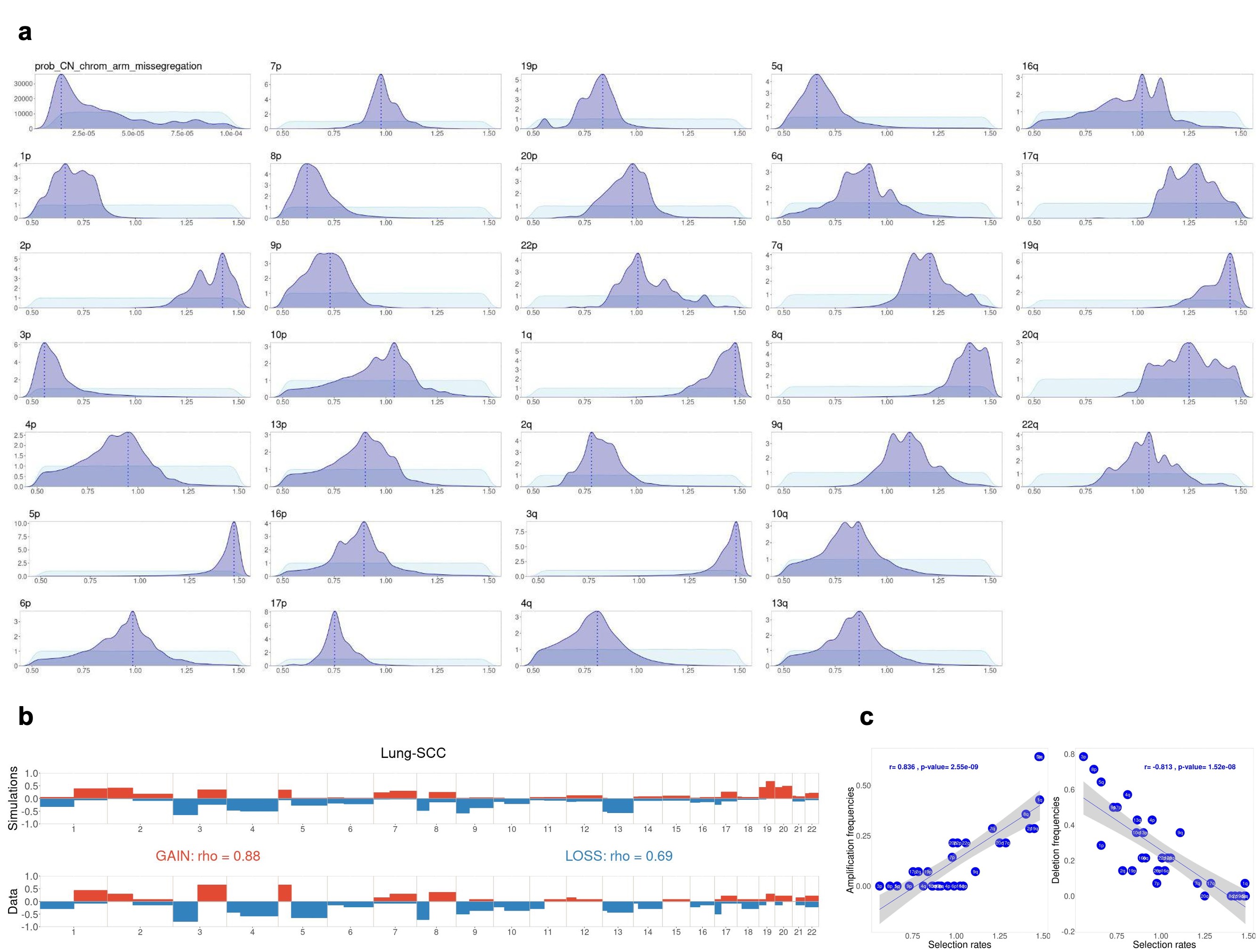

Supplement: S11 Fig — (a) Prior distribution (light blue) and posterior distribution (dark blue) from inference with ABC random forest. Broken line represents the mode in the posterior distribution for each parameter. (b) Comparison between simulations with fitted parameter (top) and gain/loss frequencies at arm level from TCGA (bottom). The simulations are computed with the posterior modes from (a). Spearman’s correlation coefficient rho between frequencies of gains (or losses) among each arm in PCAWG and simulations. (c) Correlation between inferred selection rates and amplification/deletion frequencies for individual chromosome arms. Linear regressions and p-values from Pearson correlation. (JPG) [file pcbi.1012902.s014.jpg]

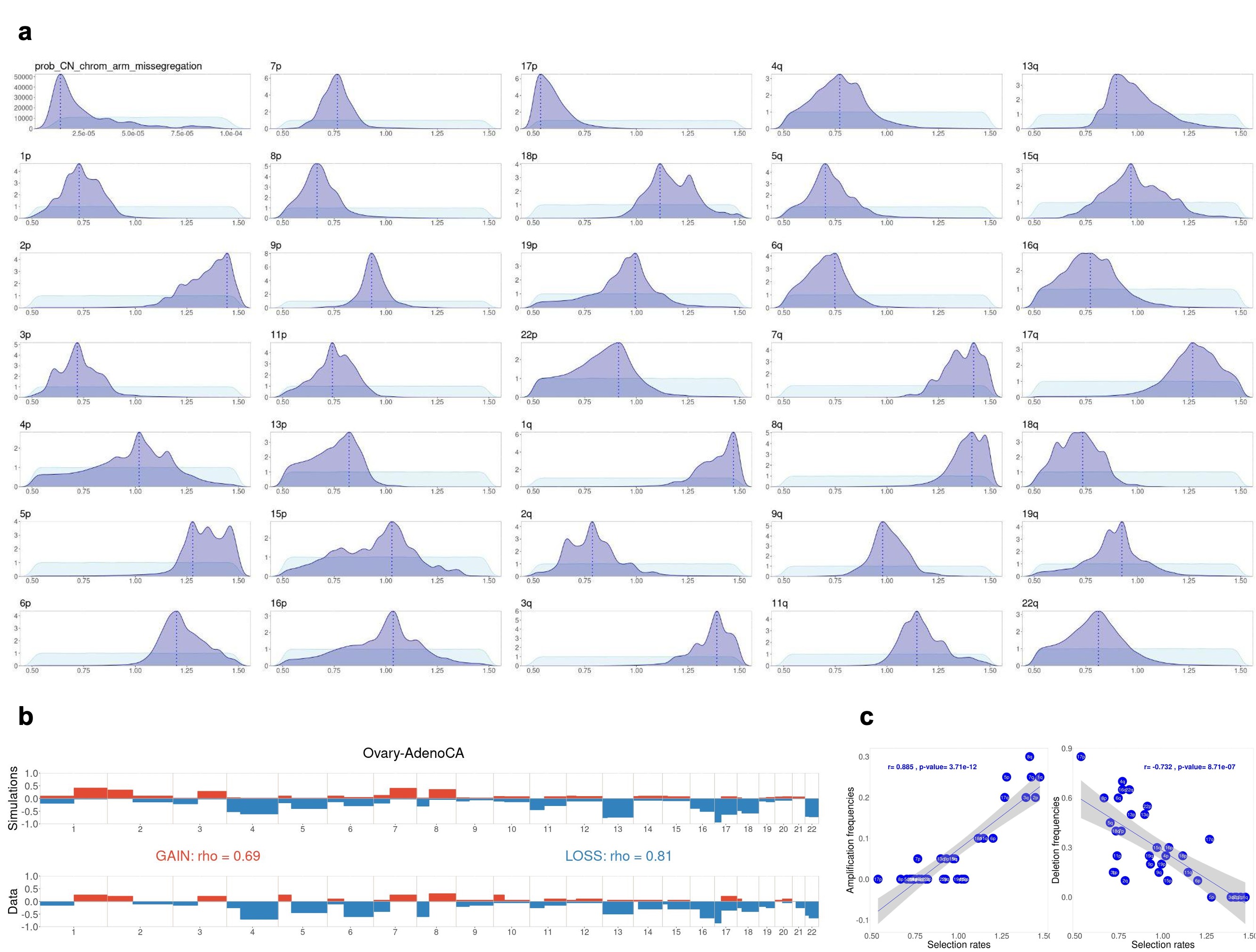

Supplement: S12 Fig — (a) Prior distribution (light blue) and posterior distribution (dark blue) from inference with ABC random forest. Broken line represents the mode in the posterior distribution for each parameter. (b) Comparison between simulations with fitted parameter (top) and gain/loss frequencies at arm level from TCGA (bottom). The simulations are computed with the posterior modes from (a). Spearman’s correlation coefficient rho between frequencies of gains (or losses) among each arm in PCAWG and simulations. (c) Correlation between inferred selection rates and amplification/deletion frequencies for individual chromosome arms. Linear regressions and p-values from Pearson correlation. (JPG) [file pcbi.1012902.s015.jpg]

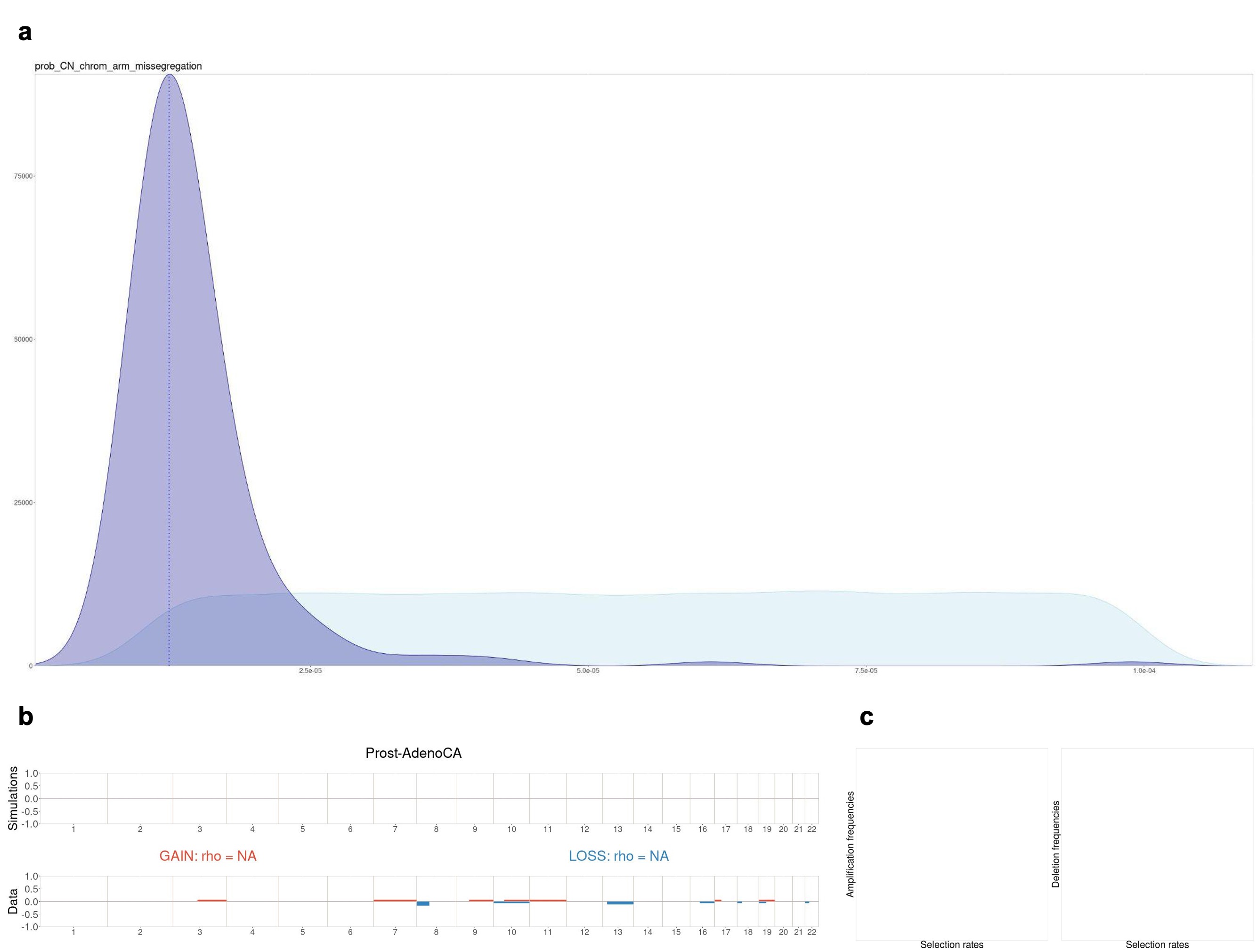

Supplement: S13 Fig — (a) Prior distribution (light blue) and posterior distribution (dark blue) from inference with ABC random forest. Broken line represents the mode in the posterior distribution for each parameter. (b) Comparison between simulations with fitted parameter (top) and gain/loss frequencies at arm level from TCGA (bottom). The simulations are computed with the posterior modes from (a). Spearman’s correlation coefficient rho between frequencies of gains (or losses) among each arm in PCAWG and simulations. (c) Correlation between inferred selection rates and amplification/deletion frequencies for individual chromosome arms. Linear regressions and p-values from Pearson correlation. (JPG) [file pcbi.1012902.s016.jpg]

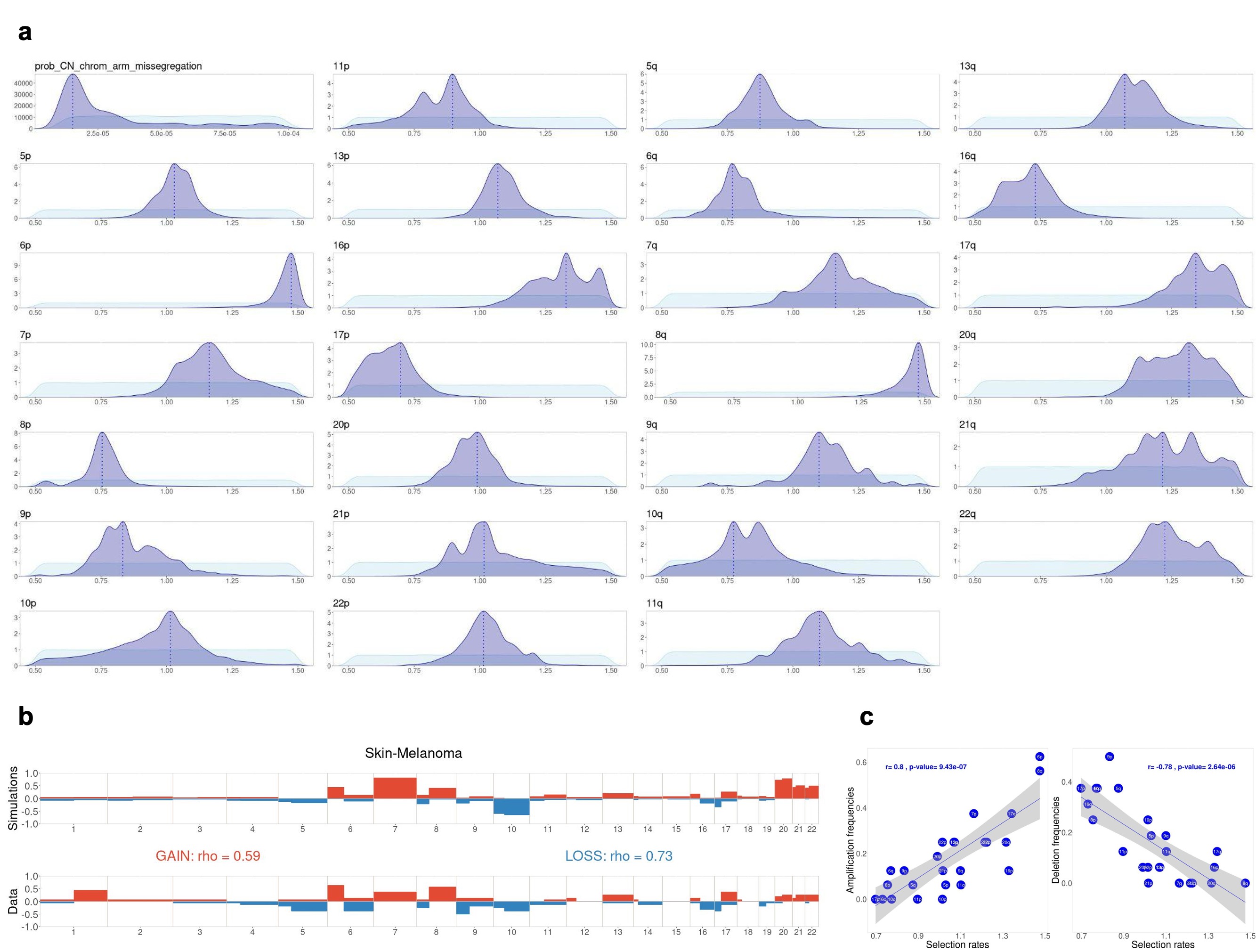

Supplement: S14 Fig — (a) Prior distribution (light blue) and posterior distribution (dark blue) from inference with ABC random forest. Broken line represents the mode in the posterior distribution for each parameter. (b) Comparison between simulations with fitted parameter (top) and gain/loss frequencies at arm level from TCGA (bottom). The simulations are computed with the posterior modes from (a). Spearman’s correlation coefficient rho between frequencies of gains (or losses) among each arm in PCAWG and simulations. (c) Correlation between inferred selection rates and amplification/deletion frequencies for individual chromosome arms. Linear regressions and p-values from Pearson correlation. (JPG) [file pcbi.1012902.s017.jpg]

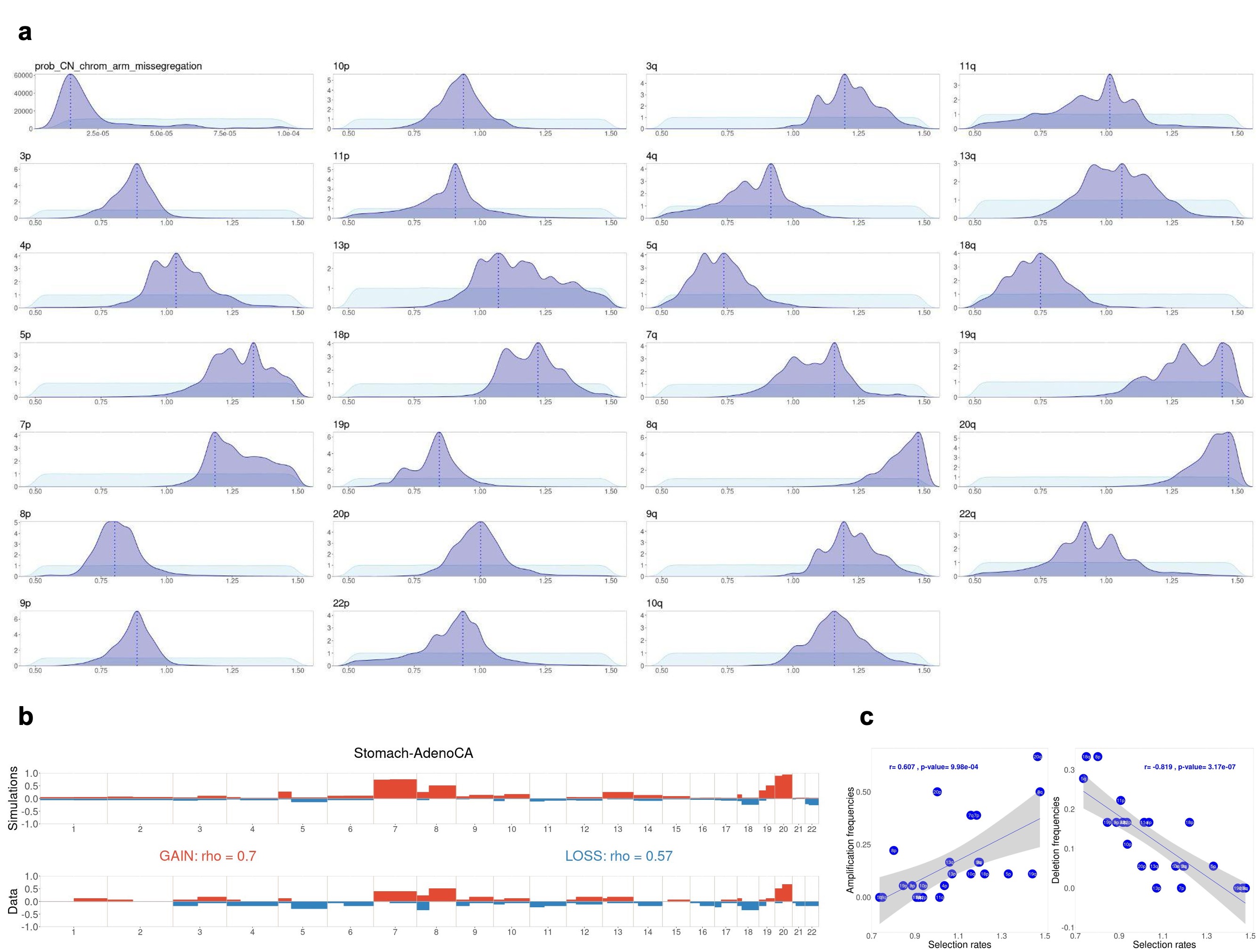

Supplement: S15 Fig — (a) Prior distribution (light blue) and posterior distribution (dark blue) from inference with ABC random forest. Broken line represents the mode in the posterior distribution for each parameter. (b) Comparison between simulations with fitted parameter (top) and gain/loss frequencies at arm level from TCGA (bottom). The simulations are computed with the posterior modes from (a). Spearman’s correlation coefficient rho between frequencies of gains (or losses) among each arm in PCAWG and simulations. (c) Correlation between inferred selection rates and amplification/deletion frequencies for individual chromosome arms. Linear regressions and p-values from Pearson correlation. (JPG) [file pcbi.1012902.s018.jpg]

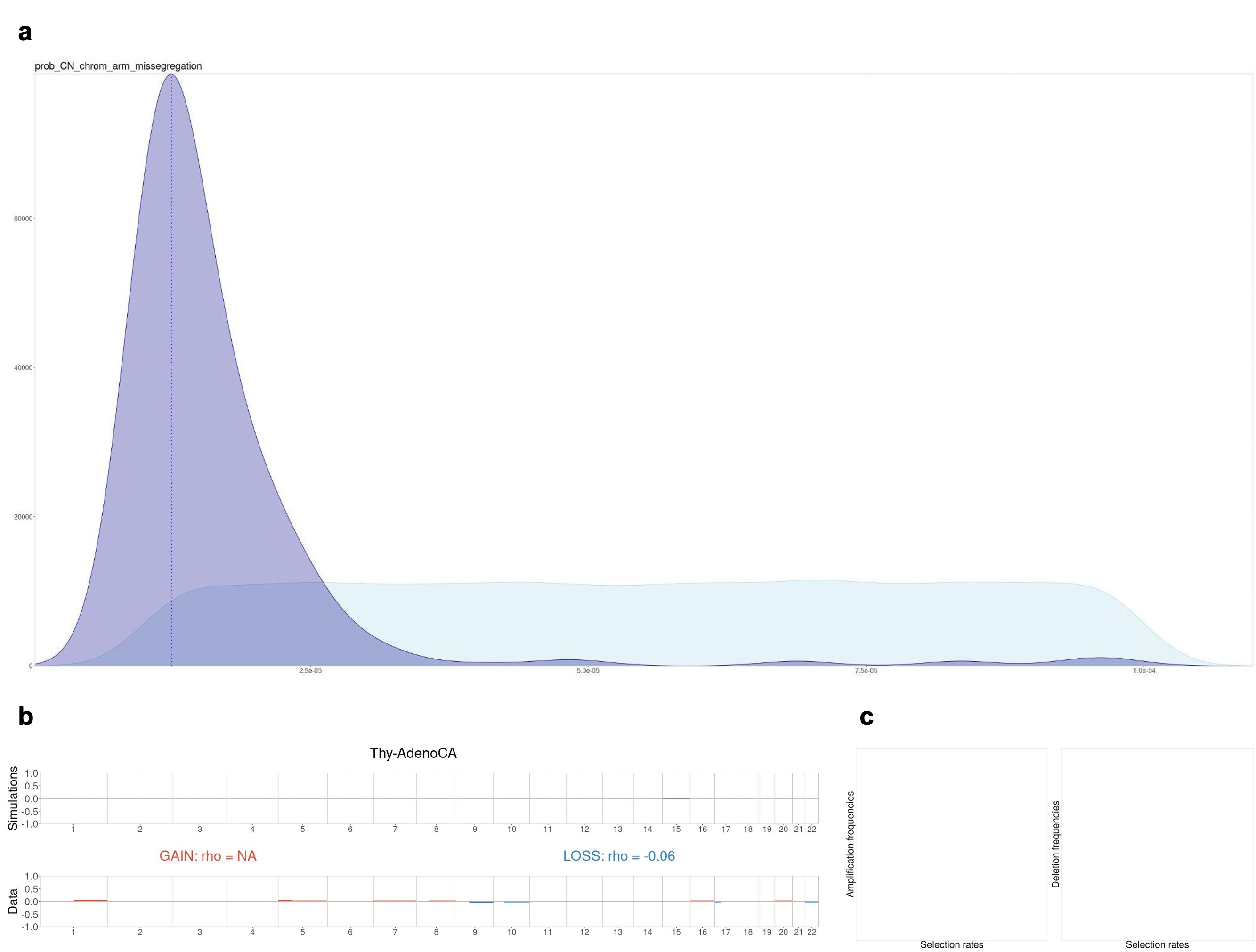

Supplement: S16 Fig — (a) Prior distribution (light blue) and posterior distribution (dark blue) from inference with ABC random forest. Broken line represents the mode in the posterior distribution for each parameter. (b) Comparison between simulations with fitted parameter (top) and gain/loss frequencies at arm level from TCGA (bottom). The simulations are computed with the posterior modes from (a). Spearman’s correlation coefficient rho between frequencies of gains (or losses) among each arm in PCAWG and simulations. (c) Correlation between inferred selection rates and amplification/deletion frequencies for individual chromosome arms. Linear regressions and p-values from Pearson correlation. (JPG) [file pcbi.1012902.s019.jpg]

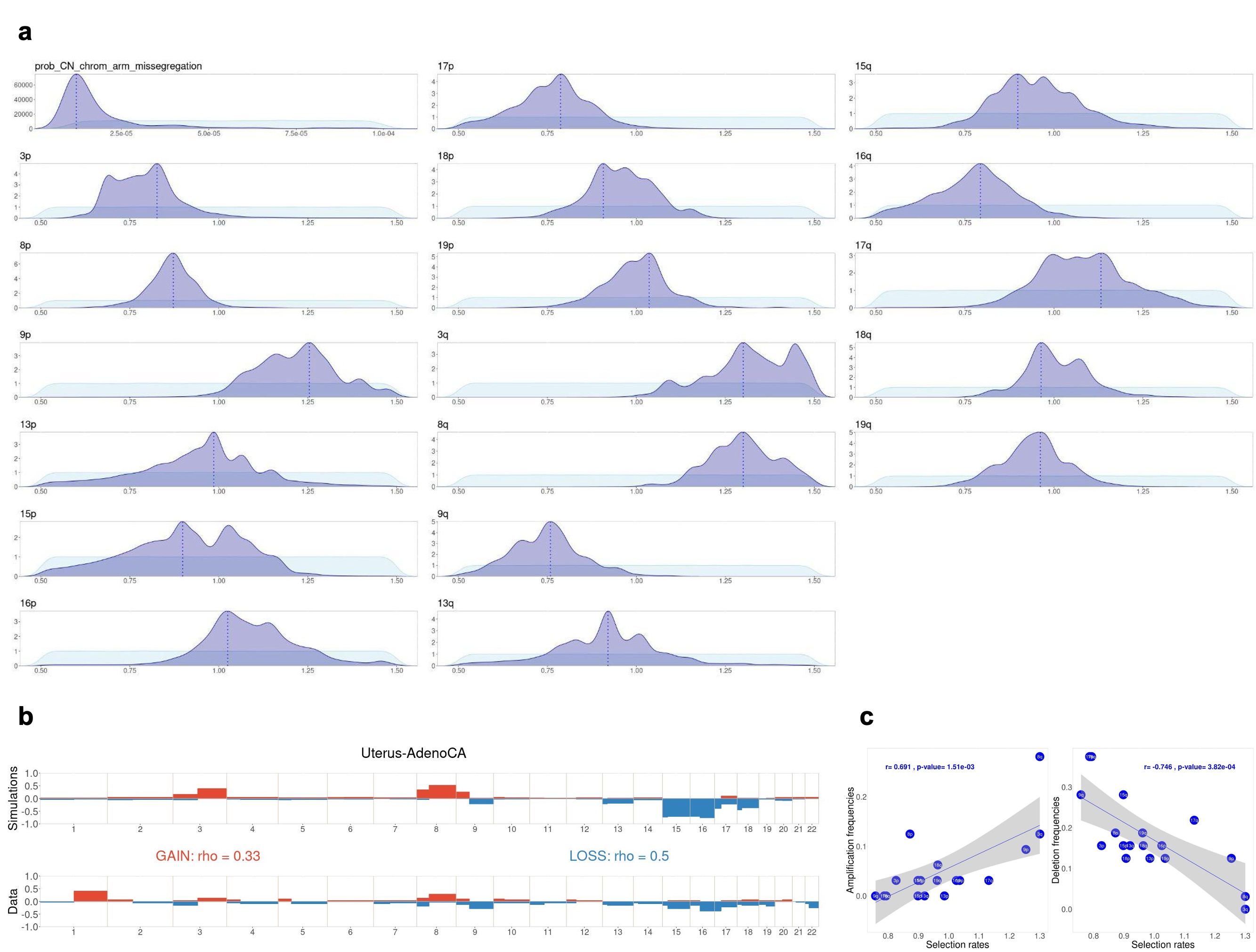

Supplement: S17 Fig — (a) Prior distribution (light blue) and posterior distribution (dark blue) from inference with ABC random forest. Broken line represents the mode in the posterior distribution for each parameter. (b) Comparison between simulations with fitted parameter (top) and gain/loss frequencies at arm level from TCGA (bottom). The simulations are computed with the posterior modes from (a). Spearman’s correlation coefficient rho between frequencies of gains (or losses) among each arm in PCAWG and simulations. (c) Correlation between inferred selection rates and amplification/deletion frequencies for individual chromosome arms. Linear regressions and p-values from Pearson correlation. (JPG) [file pcbi.1012902.s020.jpg]

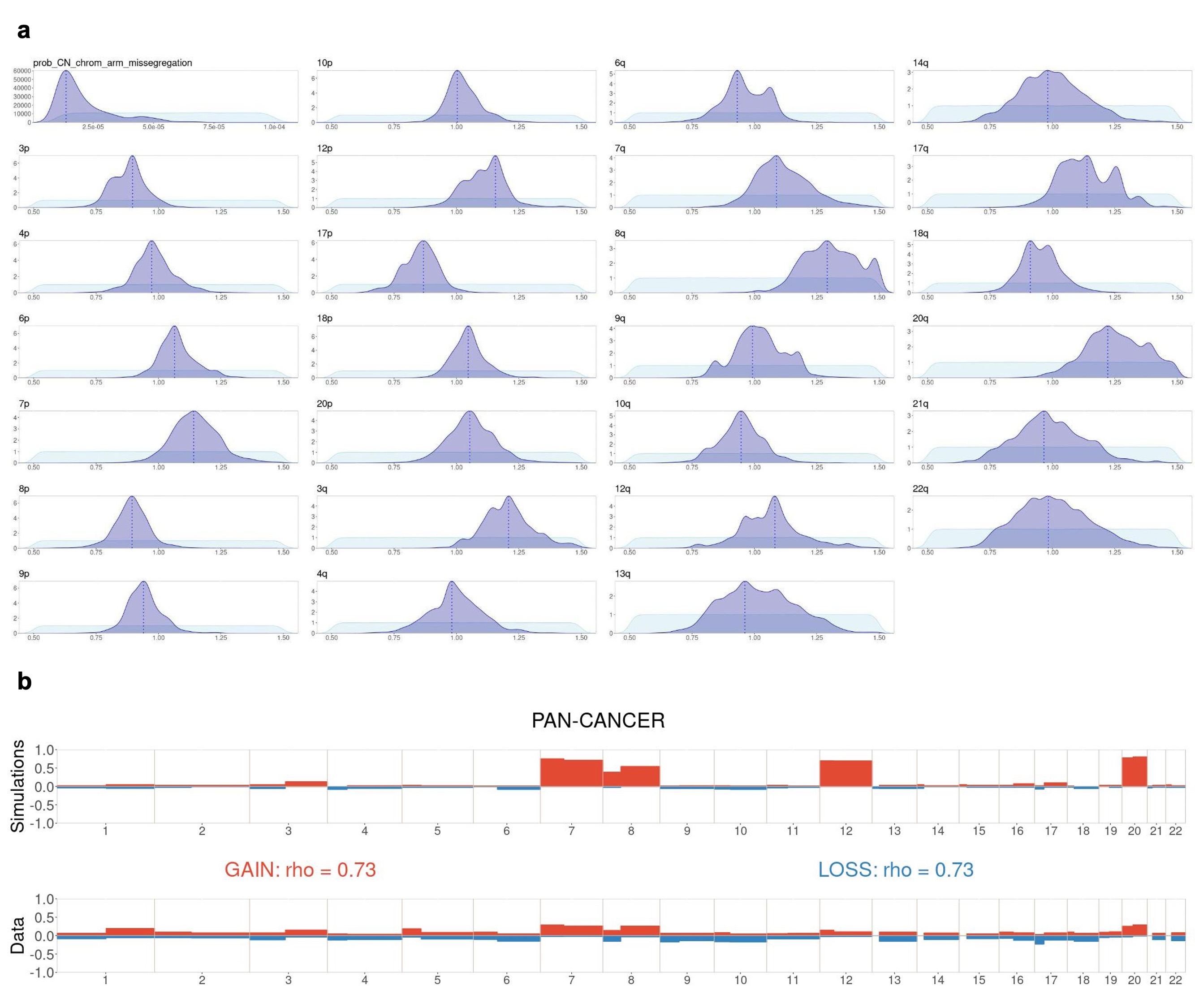

Supplement: S18 Fig — (a) Prior distribution (light blue) and posterior distribution (dark blue) from inference with ABC random forest. Broken line represents the mode in the posterior distribution for each parameter. (b) Comparison between simulations with fitted parameter (top) and gain/loss frequencies at arm level from TCGA (bottom). The simulations are computed with the posterior modes from (a). Spearman’s correlation coefficient rho between frequencies of gains (or losses) among each arm in TCGA and simulations. (JPG) [file pcbi.1012902.s021.jpg]

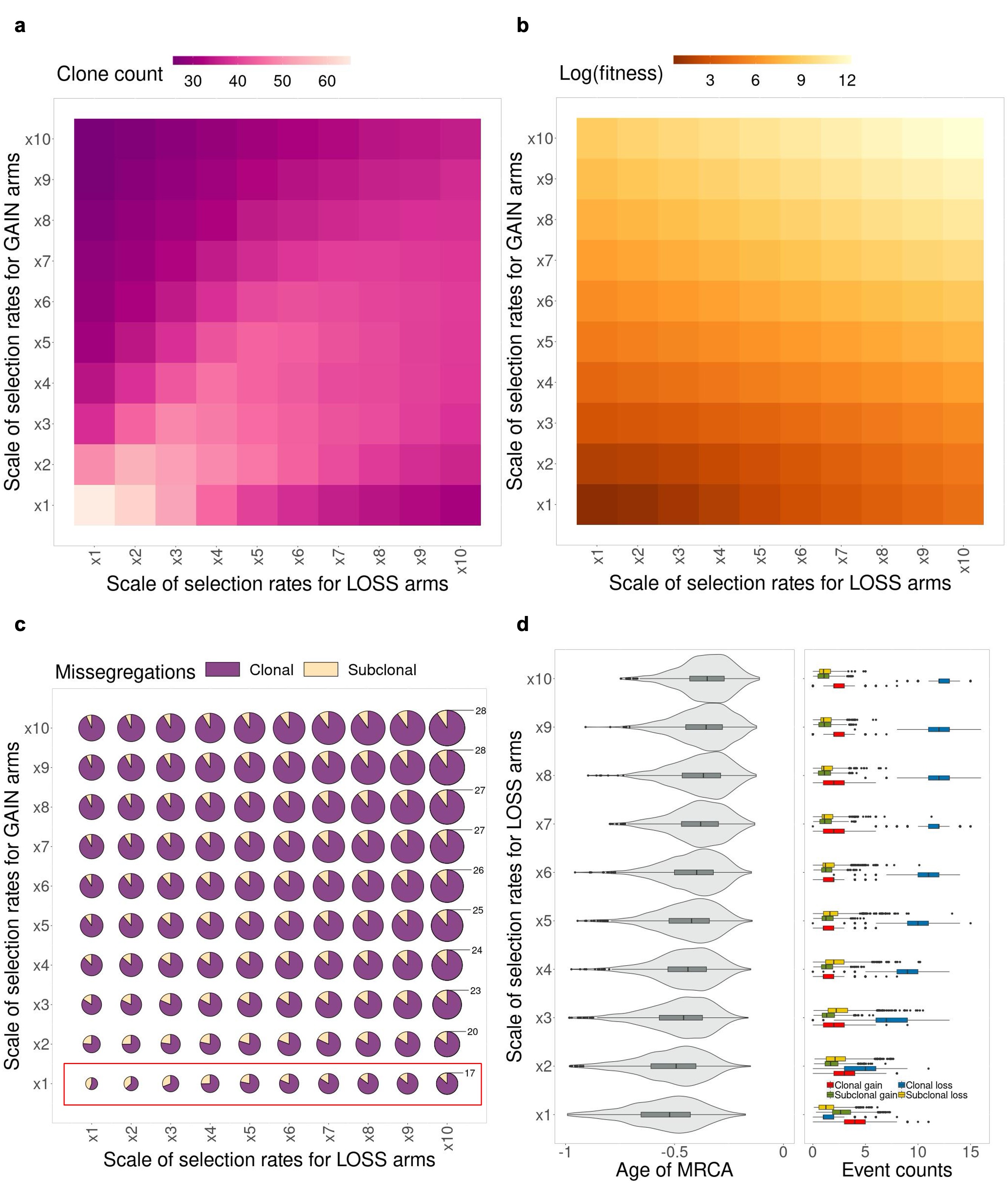

Supplement: S19 Fig — (a–c) Impact of varying parameters on clone count (a), average cell fitness (b), and average count of clonal and subclonal missegregations (c) (size of circles indicates the total missegregation counts). (c) MRCA age and average missegregation counts, grouped based on clonality (clonal/subclonal) and type (gain/loss), as selection rates for LOSS arms increase (variables correspond to highlighted segment in (c)). (JPG) [file pcbi.1012902.s022.jpg]

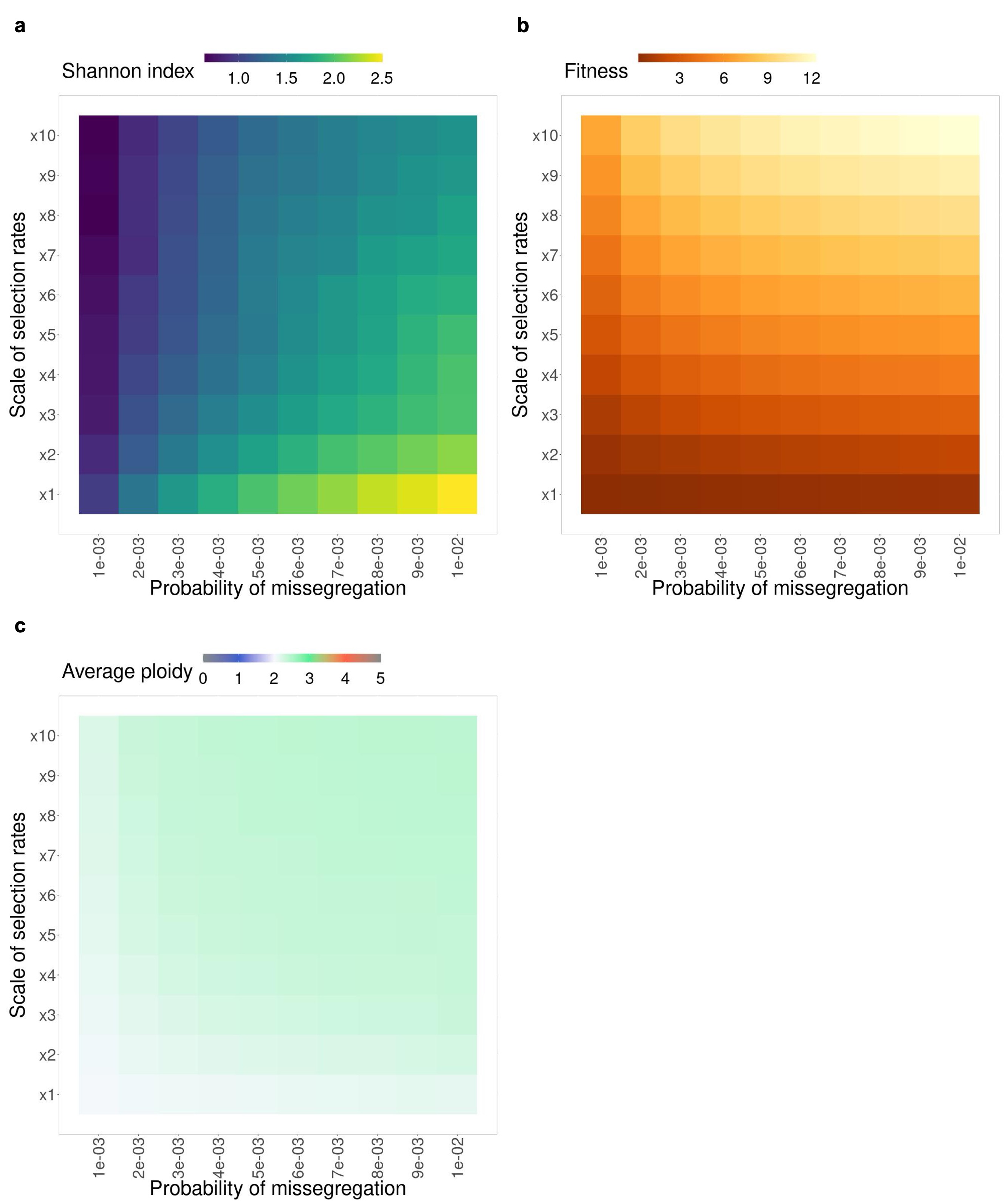

Supplement: S20 Fig — Impact of varying parameters on average Shannon diversity index (a), average fitness (b), and average ploidy in sample (c). (JPG) [file pcbi.1012902.s023.jpg]

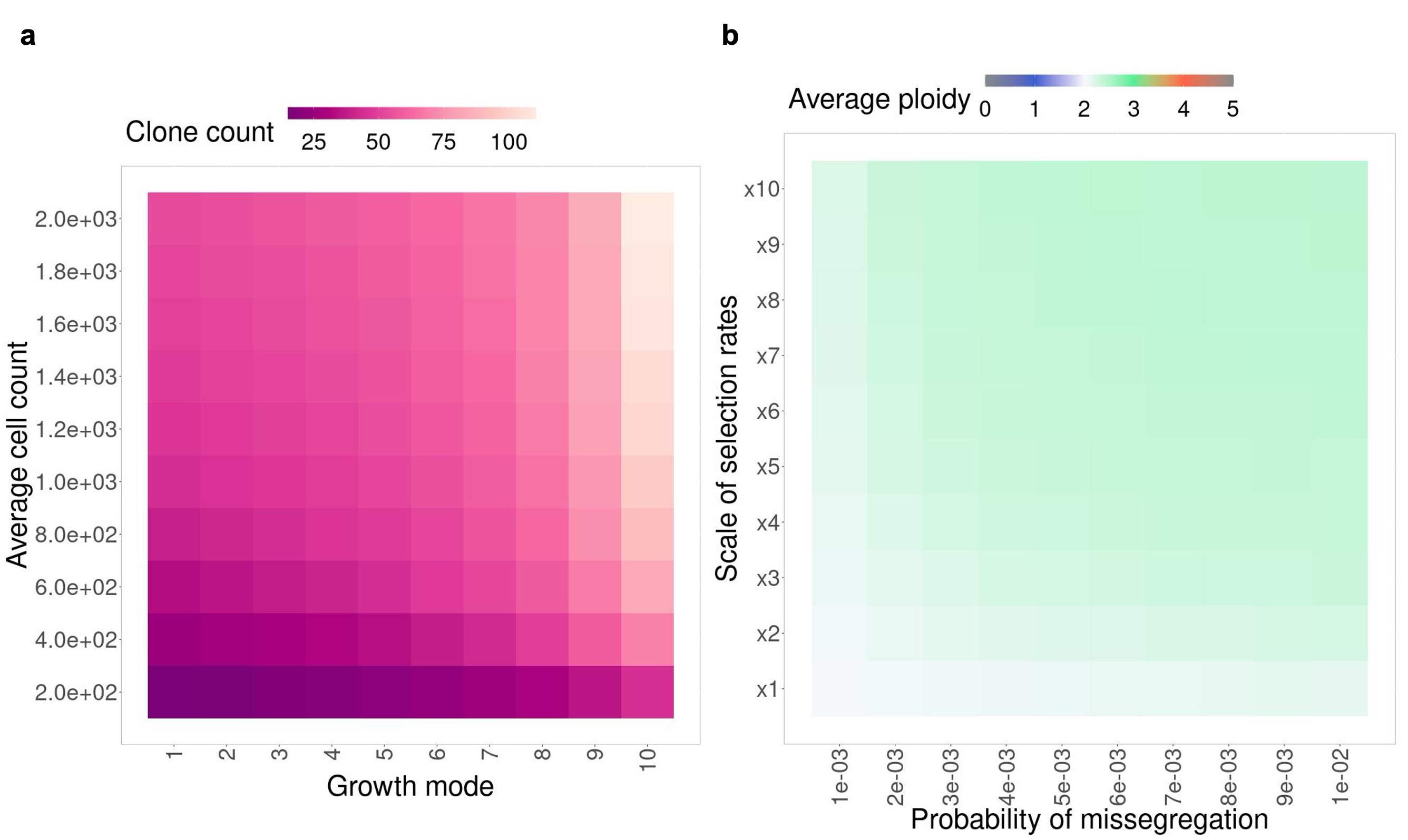

Supplement: S21 Fig — Impact of varying parameters on clone count (a), and average ploidy in sample (b). (JPG) [file pcbi.1012902.s024.jpg]

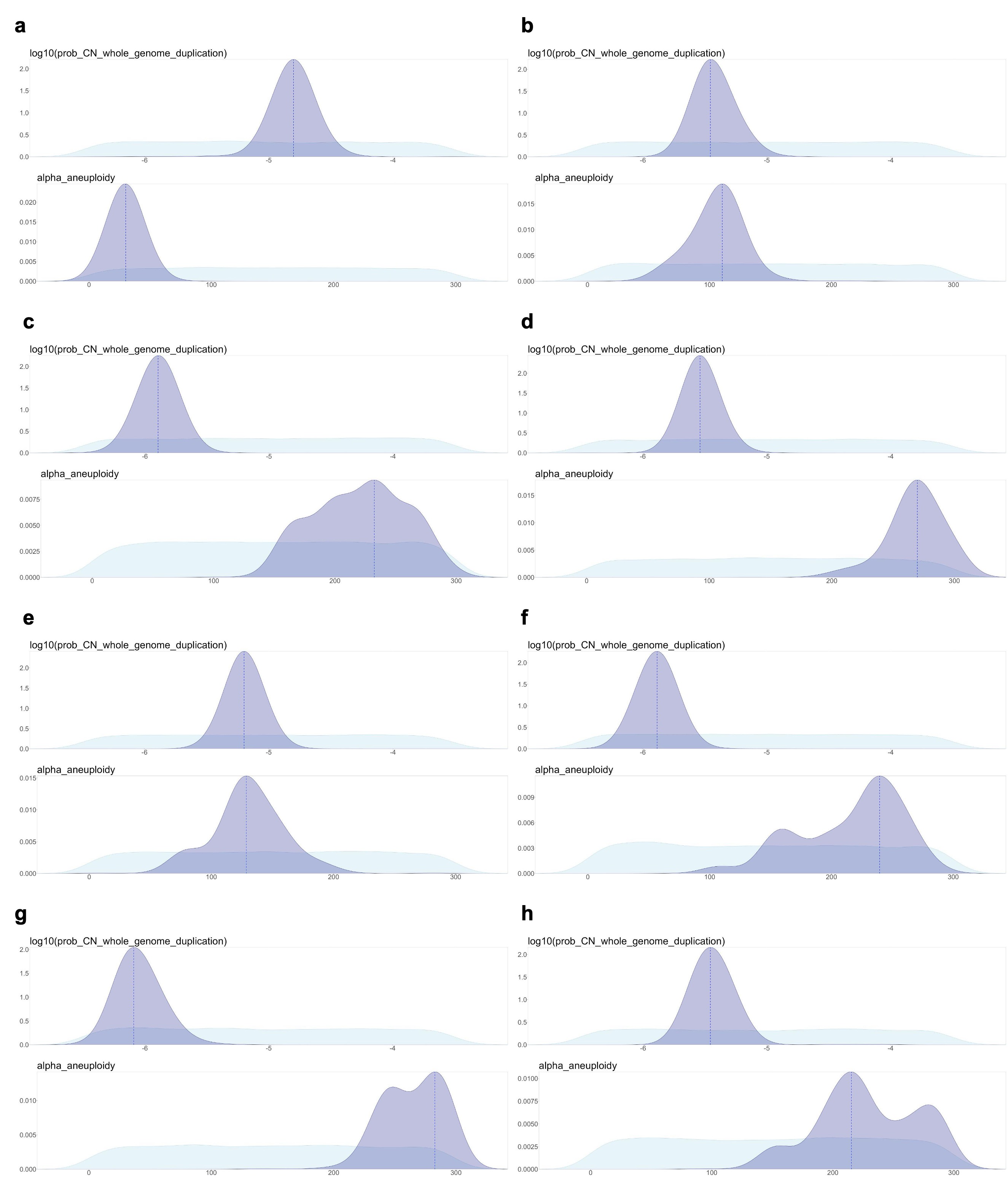

Supplement: S22 Fig — Inference of WGD probability and WGD-aneuploidy rate in individual PCAWG cancer types. Prior distribution (light blue) and posterior distribution (dark blue) from inference with ABC random forest, for Breast-AdenoCA (a), Cervix-SCC (b), CNS-GBM (c), ColoRect-AdenoCA (d), Head-SCC (e), Kidney-ChRCC (f), Kidney-RCC (g), and Liver-HCC (h). Broken line represents the mode in the posterior distribution for each parameter. (JPG) [file pcbi.1012902.s025.jpg]

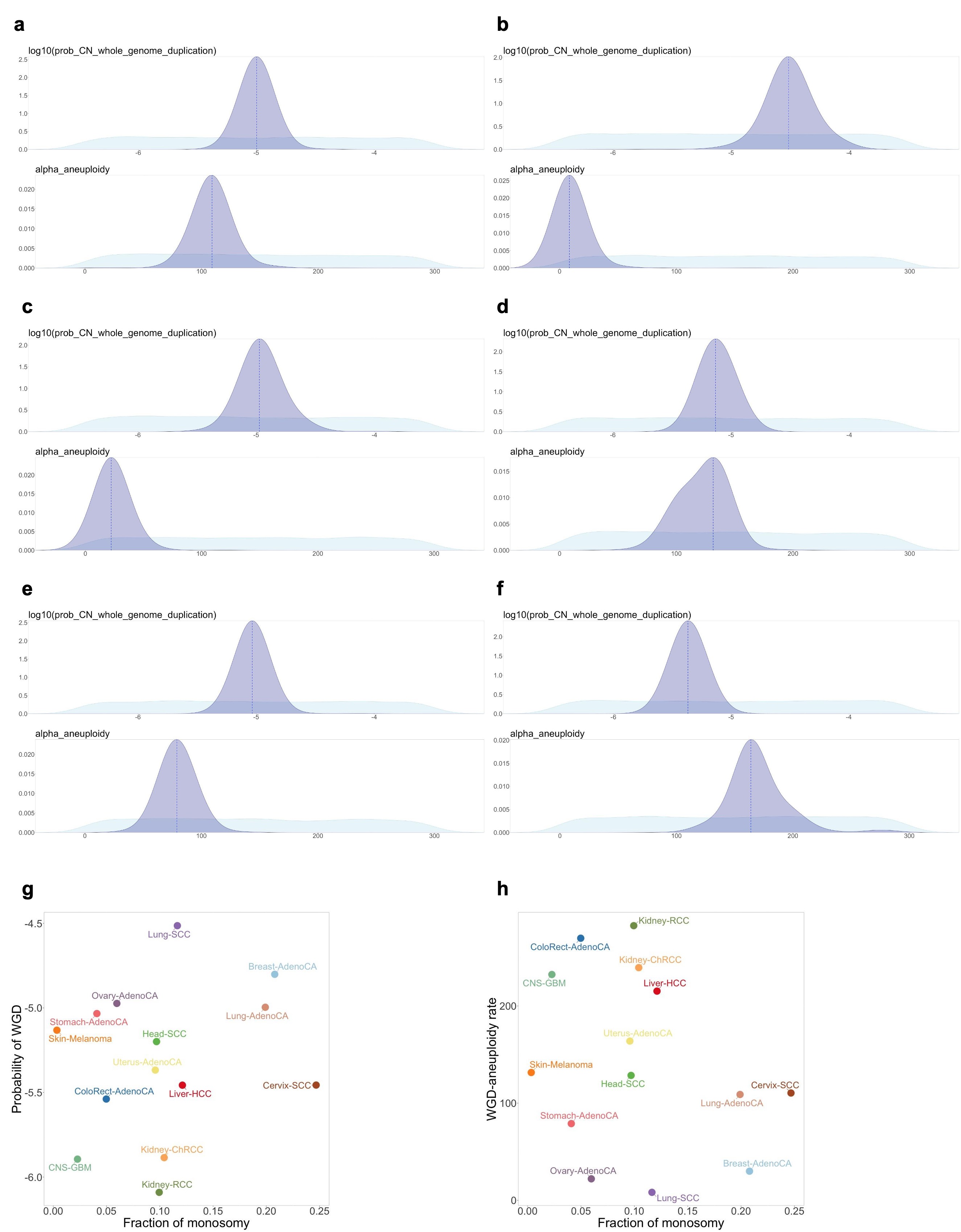

Supplement: S23 Fig — (a–f) Prior distribution (light blue) and posterior distribution (dark blue) from inference with ABC random forest, for Lung-AdenoCA (a), Lung-SCC (b), Ovary-AdenoCA (c), Skin-Melanoma (d), Stomach-AdenoCA (e), and Uterus-AdenoCA (f). Broken line represents the mode in the posterior distribution for each parameter. (g and h) Comparisons between average genomic fraction of monosomy in non-WGD samples and inferred WGD probability (g) and WGD-aneuploidy rate (h) for each cancer type. (JPG) [file pcbi.1012902.s026.jpg]

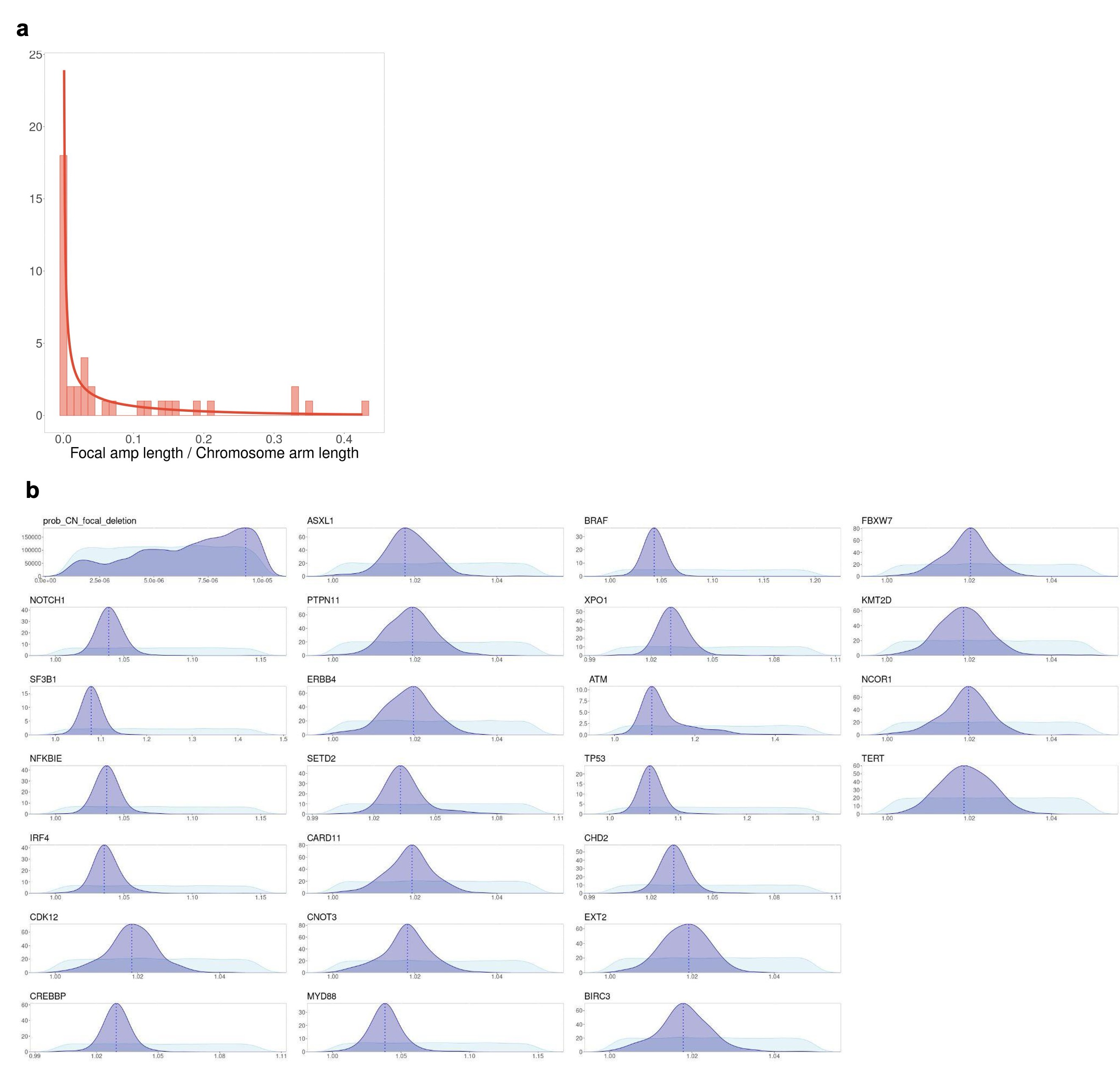

Supplement: S24 Fig — (a) Ratios of focal amplification lengths over corresponding chromosome arm lengths are fitted with a Beta distribution. (b) Prior distribution (light blue) and posterior distribution (dark blue) from inference with ABC random forest. Broken line represents the mode in the posterior distribution for each parameter. (JPG) [file pcbi.1012902.s027.jpg]
