# Supplementary material for: CINner: Modeling and simulation of chromosomal instability in cancer at single-cell resolution
Source: PLoS Comput Biol. 2025 Apr 3;21(4):e1012902. doi: 10.1371/journal.pcbi.1012902 (PMC11990800; doi:10.1371/journal.pcbi.1012902)
Supplement: Attachment — Submitted filename: Revision - Response to Reviewers.pdf [file pcbi.1012902.s030.pdf]

Reviewer #1: CINner: modeling and simulation of chromosomal instability in cancer at single-cell resolution

The manuscript by Dinh and colleagues presents a comprehensive mathematical framework, CINner, for modelling chromosomal instability (CIN) during cancer evolution. The manuscript is well-structured and provides detailed insights into the capabilities of CINner, its applications, and its potential impact on the oncology community.

We thank the Reviewer for the suggestions, which have allowed us to clarify and strengthen our manuscript.

Major comments:
While the manuscript is detailed, some sections are dense and could benefit from simplification. For example, the section on the different selection models (pages 4-5) could be simplified by summarizing in bullet-point format.

We have rewritten parts of the manuscript. In particular, the description of the selection models is simplified.

Pg5: "As all three models are defined upon the gene balance in a cell, which is retained after WGD, a WGD cell has the same fitness as its parental cell." This is quite a big assumption. Should be discussed further as quite a big limitation. What evidence exists that this is a small effect?

We acknowledge that this sentence in the original manuscript was misleading. We expect WGD to significantly impact a cell's evolution trajectory, as has been observed in many experimental studies (doi: 10.1038/s41588-020-0584-7, 10.1038/s41586-019-1907-7). In this paper, we focus on the increased chromosomal instability (CIN) associated with WGD (doi: 10.1038/s41586-022-04578-4, 10.1038/nature14187). Therefore, although the cell immediately after WGD would have the same fitness as its parent cell (due to the same gene balance relative to ploidy), we assume that this cell and all its progeny have much increased probabilities of missegregation and other CNA events.

*In Section 3, we show that increased missegregation probabilities result in more heterogeneous cell populations (**Fig. 3d-f**), increasing the likelihood of reaching a fitter karyotype. Therefore, as CNA probabilities in WGD cells are increasingly higher than in non-WGD cells, the WGD populations are increasingly fitter than the non-WGD populations (from bottom to top in **Fig. 4b-c**).*

WGD is often inferred to be an early event in patient-derived bulk cancer data (doi:10.1038/s41586-019-1907-7). Direct measurement of its fitness in vivo is therefore challenging, as it is difficult to infer whether the expansion of WGD cells is due to the selective advantage of the WGD event itself, or due to the increased CIN resulting from it. We note that in vitro studies have indicated that tetraploid populations require additional metabolic resources, compared to their diploid counterparts (doi:10.1038/nrm1276). As a result, in yeast, cells with

ploidy 4 were shown to have lower growth rate than cells with ploidy 2, which in turn grow slower than cells with ploidy 1 (doi:10.1038/nature14187).

*It is possible to incorporate this effect within CINner. Currently, the turnover rate is the same across different cells in the population (**Fig. 1a**). Cell populations with higher turnover rates divide and therefore expand faster. The aforementioned in vitro observations can be modelled as clone-dependent turnover rate, which increases with lower ploidy. However, the effects of this mechanism would be inseparable from the WGD-associated increase in CIN already included in the current manuscript, and therefore cannot be inferred independently from bulk in vivo data. We note that our recent single-cell data (doi:10.1101/2024.07.11.602772) shows that WGD is pervasive in many solid cancers but can also be present at low prevalence in some tumors. In the near future, we plan to adapt CINner to analyze these samples. The increased resolution at the cell level promises to aid in understanding distinct and sometimes contrasting effects of WGD during tumor development.*

We have thus changed this sentence to "WGD is an early and ongoing event in many cancers [24, 25], and is associated with an altered selection landscape [26] and chromosomal instability (CIN) [27, 28]. In this manuscript, we focus on the increased CIN resulting from subsequent mitoses after a cell acquires WGD. Because all three models are defined upon the gene balance in a cell, a cell retains the same fitness immediately after WGD. However, we assume that the cell and its progeny have significantly higher CNA probabilities during division." (lines 132-137).

Pg5: "Therefore, it is not necessary to simulate single cells in the whole population individually, and instead we focus on clones, defined as groups of cells that have identical CN and mutational characteristics." This is a sensible approach, but in the introduction you also state CINer ".. can generate tumors with sizes and karyotypes that match observations from DNA-sequencing data from real cancer samples." I think the statement in the introduction is a bit misleading as you are not directly simulating tumours with realistic sizes.

*The Reviewer is correct that although the clonal dynamics (step 1, **Fig. 1e**) in CINner simulations can be of realistic sizes (compared to real tumors), we only generate cell-specific information, specifically phylogeny, for sampled cells (step 2-3, **Fig. 1e**). The number of cells in sequencing samples are typically many orders of magnitude smaller than the number of cells in tumors, for both bulk and single-cell sequencing.*

To clarify this point, we changed the sentence in the Introduction to "It can generate the clonal dynamics for cell populations of sizes comparable to real tumors, from which the phylogeny tree and cell-specific measurements can be simulated for a subsample of cells, mimicking how DNA-sequencing data is produced." (lines 62-64)

On a related note, are the population genetic processes at the clonal level guaranteed to recapitulate the dynamics for the full system? I suppose this is captured in the phylogeny part but it is worth discussing and clarifying.

*The clonal evolution and the sample phylogeny, from steps 1 and 3 in CINner (**Fig. 1e**) capture distinct views of cancer development.*

Clonal evolution (from step 1) simulates the total cell population in forward time, where the population at each time point is characterized by distinct co-existing clones, their CN and driver mutation profiles, and their cell counts. This information contains all clones that arise during the whole process, including those that become extinct or are rare at the final time and hence unrepresented in the sample taken in step 2. Therefore, the interested users can examine the expansion and/or extinction of any given clone in a simulation.

On the other hand, the phylogeny (from step 3) captures the history of a subsample of cells taken at the final time point. It therefore represents information that is observable from a hypothetical tumor biopsy via DNA sequencing. This phylogeny can depict recent subclonal evolution, but may lack (a) a full view of the heterogeneity in the whole cell population, and (b) information about early population genetic processes, e.g. before the sample MRCA.

Depending on particular applications, the users may utilize the output from either step 1 or 3. We have added a paragraph (lines 172-184) to clarify these points.

The manuscript could benefit from a more explicit discussion of the limitations of CINner. addressing potential weaknesses and areas for future improvement. Currently only the fixing of the missegregation rate is included.

We added a paragraph in the Conclusion to discuss potential areas for modeling that are currently unsupported by CINner (lines 675-685).

It would be good to highlight possible ways to get around the nonidentifiability issues, such as using informative priors, hierarchical modelling and so forth.

*In our opinion, the nonidentifiability issue when simultaneously estimating the missegregation probability and selection rates likely doesn't depend on choices of inference model or priors. This is because bulk DNA data captures information at the clonal level (e.g., missegregations present in all or most sampled cells). However, clonal data such as frequencies of gains and losses across samples can be explained equally well with low missegregation probability and high selection rates, or high missegregation probability and low selection rates (**Fig. 3e**). Therefore, we expect that different inference frameworks would predict a wide-spread and strongly correlated posterior distribution, in which case individual parameters are difficult to interpret.*

*We think that the most promising alternative to avoid nonidentifiability is by employing single-cell (sc) DNA data, because the available measurements (such as Shannon index, clone count, subclonal data) can help pinpoint the most likely parameter region (**Fig. 3d-f**). In a recent study published in Scientific Reports (doi:10.1038/s41598-024-67842-9), we investigated how CINner can be applied to parameter inference using a combination of bulk and sc data. Leveraging the Approximate Bayesian Computation framework, we evaluated a broad set of statistics derived from sc copy number (CN) profiles and inferred phylogenies. We relied on synthetic data to*

*gauge the method's accuracy, finding that appropriate statistics enabled the precise estimation of posterior distributions for both chromosome-specific selection rates and missegregation probability (**Fig. 4**, doi:10.1038/s41598-024-67842-9). These results suggest that employing CINner on experimental single-cell data provides the means to get around the nonidentifiability issues. We have added this sentence in the Discussion: "Recently, we studied the application of CINner to single-cell data in a synthetic setting [73]. We considered a wide range of statistics based on observed CN profiles and inferred phylogeny trees from single-cell data. We found that the ABC framework can recover accurate posterior distributions not only for chromosome-specific selection parameters but also missegregation probabilities. We plan to combine this inference framework with CINner simulations to analyze currently available single-cell data [25, 53, 74]. (lines 665-670)*

Minor comments:
Pg11: "region of interest" might be better as "area of interest"

The phrase has been changed as suggested.

Reviewer #2:

Dinh et al. present an ambitious model of chromosomal instability and cancer evolution with an extensive, clear, and detailed supplementary methods section. This work is an exciting approach allowing the incorporation of multiple of mechanisms of genome alteration in simulations and will likely be a useful addition to the literature. However, the manuscript (and online vignettes) require much significant work to improve their clarity before publication to ensure the method is used by others. It would be a real loss if this work is not communicated clearly.

We thank the Reviewer for their valuable comments and suggestions.

Firstly, simple steps to aid the reviewers such as including line numbers should be undertaken as well as ensuring text in figures is large enough to be read when printed, e.g. in Figures 3 and 4.

We have included line numbers in the current manuscript.

*We have also significantly revised the figures to ensure legibility. Specifically, in **Fig. 3**, we moved some redundant plots to the Supplementary. The remaining (and enlarged) plots still capture the main observations that we make in this Section. Some legend items, such as the color codes for missegregation categories, have been enlarged to be legible when printed.*

*Likewise, **Fig. 4** and **5** have been reconfigured for legibility.*

Furthermore, significant changes including additional schematics and major improvements to existing figures should be made.

We have revised all figures in the main text and most figures in the supplementary, including improving legibility in existing figures, and adding schematics for the readers to understand the inference method and downstream analyses.

Concerns
1) The authors summarize the literature and contrast their approach with previous models. A main or supplementary table/figure detailing the differences in capabilities between the various previous models and CINer would be a useful addition. This table could also include potentially desirable features of copy number simulations that neither CINer nor other algorithms demonstrate – e.g. allele-specific copy number aberration simulation.

*We create **Supplementary Table 2**, which list the capabilities of CINner and previous algorithms. We include a new paragraph in the Discussion to explore future directions in expanding CINner, and the challenges to overcome (lines 675-685).*

2) Figure 1 could be much improved and/or split into multiple main and supplementary figures. Given the computational audience of the journal, particular care should be taken to clearly demonstrate SNVs and the five distinct CNA mechanisms incorporated by CINer. I would suggest individual simple schematics featuring cartoon chromosomes for each followed by a by visualisations of CINer's internal representation (current Figure 1b). Care should also be taken

regarding the description of each mechanism. The legend for 1b states "Chromosome arm missegregation misplaces a random chromosome arm", which does not adequately communicate the separate effects on the two daughter cells.

*We have completely overhauled **Fig. 1**. The new figure should be more legible and easier to understand. In particular, **Fig. 1b** is replaced by cartoon figures showing in detail how CINner updates the progeny's CN and driver mutation profiles.*

Each homolog (or strand) of a chromosome is stored as a vector in CINner, where each number is the CN of a bin. A diploid cell consists of two vectors for each chromosome, each vector containing 1 across all bins. We included cartoon figures representing the result of different CNA mechanisms and driver mutations, with rewritten descriptions in the legend.

Figure 1c is also difficult to interpret with unreadable text and could be much improved. One issue is that the selection model is for chromosome arms, yet the reader is left to identify that arms are represented in the model only by the differences in selection indicated below each heatmap representation of chromosomes 2 and 3.

*We have redrawn **Fig. 1c-d**, with increased font sizes and simplified cartoons. We included vertical solid lines to represent centrosomes, which separate the two chromosome arms, in cartoons for both the total Copy Number profiles and selection rates.*

The legend for Figure 1e is inadequate. What do the vertical dashed arrows indicate. In addition, could the authors describe the various outputs of the method in detail in the supplementary or vignettes?

In the original figure, the vertical dashed arrows indicated the data that can be output from each step in CINner.

*We have redrawn the schematic to better illustrate this point. The new legend for **Fig. 1e** is "Left: schematics of the simulation algorithm, divided into four consecutive main steps. Right: data available to be computed in each step (see Supplementary Note 2). CINner can complete prematurely if the later steps are not necessary, depending on the data requested by the user." (lines 212-214)*

Furthermore, at the end of each Section 2.2-2.5 in the Supplementary Notes, we added sentences to explain available data from each step. We also explained the output data in detail on Github (Readme file at https://github.com/dinhngockhanh/CINner, which points to an introductory vignette at https://dinhngockhanh.github.io/CINner/CINner.html).

3) Many design choices are justified by "efficiency" – what are the computational demands of simulating the growth of tumor with this algorithm?

There are two main numerical techniques that we use in CINner to increase efficiency:

- *The "down-up-down" algorithm, in which the clonal evolution in the total cell population is simulated in forward time (step 1, **Fig. 1e**), then the phylogeny for a cell*

*sample is simulated in backward time (step 3, **Fig. 1e**), and finally neutral mutations and CNAs are imposed on the sample phylogeny (step 4, **Fig. 1e**).*

- *Clonal evolution (step 1, **Fig. 1e**) is simulated using the tau-leaping algorithm, which approximates the exact Gillespie algorithm. Instead of simulating individual cell divisions/deaths as in the Gillespie algorithm, the tau-leaping algorithm simulates the total event counts in each clone during a small time step τ, updates the system state, then proceeds to the next time point.*

The use of these two techniques means that CINner runtime does not scale with the total tumor population, which can consist of millions to billions of cells. Instead, the runtimes for steps 1 and 3 (which are the most computationally demanding in CINner) are as follow:

- *Clonal evolution (step 1, **Fig. 1e**): the computational demand depends on both the clone count and the number of time steps from start to finish. When the probabilities of CNA and driver mutations increase, the number of clones also increases. Conversely, reducing time step τ, while resulting in closer approximation of Gillespie, also increases the numerical cost.*
- *Sample phylogeny (step 3, **Fig. 1e**): the runtime scales with sample size.*

We have added the following paragraph: "The use of the "down-up-down" strategy and tau-leaping algorithm allows for significantly reduced runtime of CINner, compared to directly simulating the branching process for the whole population and then extracting the phylogeny only for the sampled cells. The runtime of simulating the clonal evolution (step 1, Fig. 1e) scales with the number of clones and the number of time steps. The clone count increases with higher CNA and driver mutation probabilities. The number of time steps increases inversely with the step size selected for the tau-leaping algorithm. Meanwhile, the computational cost of simulating the phylogeny (step 3, Fig. 1e) scales with the number of sampled cells, which is typically of magnitudes smaller than the population size." (lines 163-170)

4) The parameters used by the model and parameter estimation program referenced on page 7 are insufficiently explained in the main text and figures. Schematic figure(s) briefly describing these as well as the analyses and their motivation performed in Figure 2 should be included to aid reader comprehension.

*We created a schematic for the inference and analysis of cancer type-specific chromosome arm selection parameters (**Fig. 2a**). The cartoon gives an overview of the main CINner parameters of interest (chromosome arm selection rates), which in the inference are sampled from uniform prior distributions. We then use Approximate Bayesian Computation to find the posterior distributions, by comparing the gain/loss frequencies from CINner simulations with each parameter set to the gain/loss frequencies from a data cohort. For the downstream analyses, we first classify each arm as a GAIN or LOSS arm, if the mean posterior selection rate is more or less than 1, respectively. We then summarize the inference results with the count and mean selection rate across all GAIN and LOSS arms. Finally, we validate these results by comparing the summary statistics with tissue-specific WGD proportions (**Fig. 2h-j**) or the Charm scores (**Fig. 2k-l**).*

5) The PCAWG and TCGA acronyms are introduced without explanation. In addition, the

numbers of cases analyzed should be made clear in supplementary tables and figure legends. The authors could also consider including the datasets and number of samples analyzed in the abstract.

We included the introduction of the Pan-Cancer Analysis of Whole Genomes (PCAWG) (line 241) and The Cancer Genome Atlas (TCGA) (line 306).

*We also created **Supplementary Table 1**, which lists the numbers of non-WGD and WGD samples, employed respectively for the inference of chromosome-arm selection parameters (**Fig. 2**) and WGD and associated CIN (**Fig. 4**). We added sample sizes in the legends for relevant figures (**Fig. 2b-d**, **2k-l**, **5**, **Supplementary Fig. S1-S18**, **S22-S24**).*

Finally, we included descriptions of the datasets with sample counts used in our study in the Abstract.

6) The authors should make it clear that tissue and tumor-type specific patterns of aneuploidy are well known and described in the literature with appropriate references. In addition, care should be taken over language. For instance, the sentence "Cancer cells that have amplified GAIN arms are more selective" should be rewritten.

We included the references for previous cancer type-specific aneuploidy studies in the following sentence: "We have shown that specific cancer types exhibit a wide discrepancy in chromosome arm driver count, the potency of these arms, and the distribution of GAIN and LOSS arms among them, as has been previously reported extensively [9, 20, 22, 26, 37, 48]." (lines 354-357)

*We have rewritten parts of this Section to make clear that the analyses (**Fig. 3**) consist of parameter studies in CINner. Specifically, the sentence "Cancer cells that have amplified GAIN arms are more selective…" has been rewritten as "In CINner's framework, amplifications of GAIN arms are more advantageous as the selection parameters of these arms increase. During simulated evolution, cancer cells with these amplifications acquire higher fitness rates and are more likely to expand." (lines 361-363)*

7) Given that the CINer is estimating selection parameters from individual cancer types it would be interesting to see a comparison between the CHARM scores that are estimated from pan-cancer SNV data and their own cancer-type specific scores. For example, for cancer types for which there are sufficient tumors in both PCAWG and the TCGA, could selection parameters be inferred from one dataset and examined on the other as well as being investigated with CHARM scores? One might expect that the cancer-type specific scores inferred by CINer would outperform the pan-cancer CHARM scores.

We are not aware of any cancer type-specific studies using Charm scores similar to the pan-cancer analysis by Davoli et al. This is probably due partly to the significant numbers of both cancer samples and genes required to compute arm scores that can predict gain/loss frequencies with adequate statistical power. In this work, the authors study 17921 genes in 8207 tumor samples, predict the role of each gene (as TSG, oncogene, or essential gene) based on observed patterns across $\sim 10^6$ *mutations, then compose one score for each arm or chromosome*

(doi:10.1016/j.cell.2013.10.011). Currently available tissue-specific data typically contains much fewer samples.

Furthermore, PCAWG was a study of data combined from TCGA and the International Cancer Genome Consortium (ICGC) (doi:10.1038/s41586-020-1969-6). Therefore, we cannot perform cross-validation using these data.

*Because of these considerations, we can only directly compare our results to the Charm scores for pan-cancer data (**Fig. 2k-l**). Moreover, after we infer selection parameters, we compare them to cancer type-specific WGD proportions. Because the inference uses only non-WGD samples, it is blind to the WGD prevalence. Therefore, that the selection parameters can predict WGD proportions (**Fig. 2h-j**) serves as indirect validation of our results, and implies the biological relevance of our inferred selection parameters.*

8) Could the authors clarify their meaning when they say "For instance, the genomes of WGD squamous cell lung carcinoma are significantly altered, but not at a substantially higher level than non-WGD tumours". How is the word significantly being used here? When examining figure 4a the FGA in the WGD samples looks very different to the non-WGD samples, could the authors comment?

In this paragraph, we meant to point out that while the FGA in WGD sample in some cancer types (e.g., Lung-SCC) is not much higher than the FGA in non-WGD samples, other cancer types (e.g., Kidney-RCC) have low FGA in non-WGD samples but very high FGA in WGD tumors. We rewrote these sentences to clarify: "For instance, although WGD samples of squamous cell lung carcinoma (Lung-SCC) exhibit highly altered genomes, the FGA of these samples is not much higher compared to non-WGD samples. In contrast, kidney renal cell carcinoma (Kidney-RCC) has few genomic alterations and therefore low FGA in non-WGD samples, however the WGD tumors exhibit high FGA, indicating significantly increased CIN level associated with WGD." (lines 439-443)

*Regarding the considerably different FGA, or equivalently CIN, between WGD and non-WGD samples across different cancer types (**Fig. 4a**), this has been well reported in the literature with both bulk DNA data (doi:10.3389/fcell.2023.1209136, doi:10.1038/s41586-019-1907-7) and single-cell data (doi:10.1101/2024.07.11.602772). For instance, in doi:10.1158/0008-5472.CAN-21-2065, the authors found substantially higher frequencies of gains and losses in the WGD samples, compared to non-WGD samples, in every cancer type (Fig. 2a in doi:10.1158/0008-5472.CAN-21-2065), This equates to significantly higher FGA in WGD compared to non-WGD, as shown in our **Fig. 4a**. This has been linked to higher levels of missegregation errors associated with WGD, as has been observed in yeast (doi:10.1038/nature14187). Therefore, in this work we focus on modeling the probability of WGD and the WGD-aneuploidy rate, the latter of which models the increase in missegregation probabilities associated with WGD.*

9) On page 12 a sample size is specified at 1000 cells, this seems low – what are the sample sizes used for all other simulations.

*In other parameter studies (**Fig. 3a-f**, **Fig. 4b-c**), the sample size consists of the total final cell population. Because the final cell count changes significantly for different growth modes (**Fig. 3g**), to study the effect of final cell count and growth mode we fix the sample size at 1000 cells (**Fig. 3h-l**, **Supplementary Fig. 21**).*

However, we argue that larger sample sizes are not necessary for our results to be comparable to relevant data. The parameter studies are designed to illuminate our inference results using bulk data, with potential application in understanding single-cell data. On the one hand, our inference employs the frequencies of (clonal) gains and losses in bulk data. These clonal events are observed in every cell in the sample, and therefore do not depend significantly on the sample size. On the other hand, currently available single-cell datasets consist of hundreds to a few thousands of cells per sample (e.g., doi:10.1016/j.cell.2019.10.026, doi:10.1038/s41586-021-03648-3, doi:10.1038/s41586-022-05249-0). The sample size in our parameter studies is therefore relevant to these data as well.

10) The authors repeatedly mention the limitations of bulk sequencing data and the possibility of applying CINer to single cell data. I note that the some of authors have previously published papers utilizing single cell datasets. Could the authors consider including a case study of single cell data that could be examined alongside a bulk data from the same cancer type? I understand that this may be beyond the scope of this paper and that the authors may not have bandwidth for such further analysis.

CINner was indeed designed to work for both bulk and single-cell (sc) sequencing data. However, we assumed that its application to sc cohorts requires careful consideration of the large number of different statistics that can be computed from the data, which had not been explored in the literature.

Recently, we published a study in Scientific Reports which examines the use of CINner in parameter inference from both bulk and sc data (doi:10.1038/s41598-024-67842-9), including leveraging sc datasets we had previously reported (doi: 10.1038/s41586-022-05249-0, 10.1038/s41586-021-03648-3). We considered a wide range of statistics based on the observed sc CN profiles and inferred phylogeny trees, within the Approximate Bayesian Computation framework. To determine the accuracy of the results, we focused on synthetic data. We found that with the right statistics, this framework can infer accurate posterior distributions not only for chromosome-specific selection rates but also missegregation probability (Fig. 4, doi:10.1038/s41598-024-67842-9), while requiring a relatively small number of samples (Fig. 5, doi:10.1038/s41598-024-67842-9). Therefore, application of CINner to experimental sc data promises to offer much more detailed results, compared to the analyses in this manuscript.

We are planning to implement this framework to examine our larger cohorts of sc data in the near future (e.g., doi:10.1101/2024.07.11.602772). We expect this project to be labor-intensive, and therefore warrants a separate manuscript. In particular, we want to calibrate the Approximate Bayesian Computation algorithm to maximize its performance in real data. We also plan to extend the framework in doi:10.1038/s41598-024-67842-9 to also infer probabilities of chromosome-arm missegregation and whole-genome duplication from sc data, among other CNA

mechanisms. Finally, we will compare our results of missegregation probabilities and selection parameters from sc data to experimental observations. This study will require significant work.

We have added this sentence in the Discussion: "Recently, we studied the application of CINner to single-cell data in a synthetic setting [73]. We considered a wide range of statistics based on observed CN profiles and inferred phylogeny trees from single-cell data. We found that the ABC framework can recover accurate posterior distributions not only for chromosome-specific selection parameters but also missegregation probabilities. We plan to combine this inference framework with CINner simulations to analyze currently available single-cell data [25, 53, 74]." (lines 665-670)

11) In the supplementary page 8, section 1.5.2, $\lambda_d \in (0, \infty)$ (para 1):
Presumably λ_d is different for each driver gene? Or not? The sentence is ambiguous.

The next equations only seem to make sense if $\lambda_d \geq 1$.
The definitions of λ_{d-WT} and λ_{d-MUT} seem unmotivated and are unexplained. Would it not be cleaner to have a single style of parametrisation for both, for example the ONCs might have $\lambda_{d-WT} = 1$, $\lambda_{d-MUT} = \lambda_d$, if λ_d needs to be predefined as greater than 1 for some reason?

The selection rate λ_d is indeed different for each driver gene d. Furthermore, the Reviewer is correct that it should be defined as $\lambda_d \in (1, \infty)$, instead of $(0, \infty)$. We have changed this sentence to "Driver gene d has selection rate $\lambda_d \in (1, \infty)$, which can vary for different driver genes".

Our model for driver gene selection rates is based on some general assumptions of how mutations and CN changes affect cell fitness:

- A. *WT alleles of oncogenes are less selective than alleles with mutations that confer gains of function.*
- B. *Furthermore, amplifications of both mutant and WT oncogene alleles are selective. For instance, amplifications of WT oncogenes, either in linear or extra-chromosomal DNA, are observed in doi:10.1038/s41588-020-0678-2 and doi:10.1016/j.ccell.2018.03.007.*
- C. *Mutations causing loss of function in TSGs are more selective than WT alleles.*
- D. *Deletions of TSGs are also selective.*
- E. *However, once TSG alleles lose their function, they become neutral and their amplifications or deletions do not impact cell fitness.*

We wanted to define a simple framework for selection rates that reflect these observations. The cell fitness is defined as

$$s_k = \prod \lambda_{d-WT}^{2 \cdot n_{d-WT}^{[k]}/c^{[k]}} \cdot \lambda_{d-MUT}^{2 \cdot n_{d-MUT}^{[k]}/c^{[k]}} \qquad \text{(Eq. (3), page 9, Supplementary Notes)}$$

For oncogenes, if the WT allele has selection rate $\lambda_{d-WT} = 1$ as suggested, then its amplifications/deletions are neutral (i.e., s_k is unchanged as $n_{d-WT}^{[k]}$ varies), contrary to assumption B. To characterize assumptions A-B while keeping the number of parameters at minimum, we therefore define

$$\lambda_{d-WT} = \lambda_d \qquad \lambda_{d-MUT} = \lambda_d^2$$

with cancer gene-specific λ_d. This way, s_k increases with higher $n_{d-WT}^{[k]}$ or $n_{d-MUT}^{[k]}$.

In contrast, the definitions for TSG selection rates can be cleaner:

$$\lambda_{d-WT} = 1/\lambda_d \qquad\qquad \lambda_{d-MUT} = 1$$

while still reflecting assumptions C-E.

We have added these justifications for the selection rate formulae in the Supplementary Notes (page 8).

Equation (3) on the following page, defining a cell's fitness, seems not to include the non-linear effect on fitness of total loss of both viable alleles of a TSG. Is this intentional? For example, if one arm containing an ONC/TSG is lost, and the corresponding on the remaining arm is mutated, is the combined model's estimate of fitness realistic? Are the nonlinear fitness effects that occur when LOH exposes a single mutated copy of a gene modelled adequately here?

Our underlying assumptions for the driver gene selection model, listed in the previous answer, are not universally true for all genes. In particular, fitness of TSGs depends on how their expression level affects cancer cells (Fig. 1, doi:10.1038/nature10275). For TSGs obeying the two-hit model, one allele being inactivated via loss or mutation does not affect cell fitness, while the inactivation of both alleles induces cancer. For haploinsufficient TSGs, one inactivated allele is already beneficial for the cancer cell, and the inactivation of both alleles further increases its fitness. Yet for TSGs with quasi-sufficiency and obligate haploinsufficiency, the inactivation of one allele is beneficial but the inactivation of both alleles makes the cell nonviable. Furthermore, the behavior of each TSG can also depend on the tissue and the cell's genomic context (Fig. 2, doi:10.1038/nature10275).

In this paper, we assume haploinsufficiency for TSGs. A normal cell (i.e. ploidy $c^{[k]} = 2$) with 2 WT alleles of TSG d (i.e. $n^{[k]}_{d-WT} = 2$, $n^{[k]}_{d-MUT} = 0$) has fitness

$$s_k^{[diploid]} = \lambda^2_{d-WT} \cdot \lambda^0_{d-MUT} \cdot S = \frac{1}{\lambda_d^2} \cdot S$$

where S denotes the contribution of other driver genes to the cell fitness. If one of the alleles is lost, the new cell's fitness is

$$s_k^{[1st\ hit]} = \lambda^1_{d-WT} \cdot \lambda^0_{d-MUT} \cdot S = \frac{1}{\lambda_d} \cdot S = \lambda_d \cdot s_k^{[diploid]}$$

which is higher than the parent cell, because $\lambda_d > 1$.
Alternatively, if one allele is mutated in the normal cell, the fitness effect is the same:

$$s_k^{[1st\ hit]} = \lambda^1_{d-WT} \cdot \lambda^1_{d-MUT} \cdot S = \frac{1}{\lambda_d} \cdot 1 \cdot S = \lambda_d \cdot s_k^{[diploid]}$$

Finally, if a cell has one lost allele and one mutated allele, the fitness is higher yet:

$$s_k^{[2nd\ hit]} = \lambda^0_{d-WT} \cdot \lambda^1_{d-MUT} \cdot S = S = \lambda_d^2 \cdot s_k^{[diploid]}$$

Note that this fitness would be the same if the cell alternatively loses both alleles:

$$s_k^{[2nd\ hit]} = \lambda^0_{d-WT} \cdot \lambda^0_{d-MUT} \cdot S = S = \lambda_d^2 \cdot s_k^{[diploid]}$$

or has both alleles mutated:

$$s_k^{[2nd\ hit]} \quad = \lambda_{d-WT}^0 \cdot \lambda_{d-MUT}^2 \cdot S = S = \lambda_d^2 \cdot s_k^{[diploid]}$$

*Although we observe that this simple model works relatively well for CLLE-ES (**Fig. 5**), we expect that its application to other cancer types and data cohorts would require better knowledge of the behavior of individual driver genes, especially which of the three categories listed above that each TSG belongs to in each cancer type. We are not aware of any such data, as of now. However, we note that interested readers can experiment with changing the selection rate formulae in CINner relatively easily, and we added a suggestion in the Supplementary Note to point them to function CINner_selection_rate.r in CINner's R folder.*

Minor points:
The online Readme has a spelling error "Direct Library Preperation+ (DLP+)."

We fixed the typo in the online Readme.

Reviewer #3:

The authors proposed CINner, a framework for modeling chromosomal instability during cancer evolution. I have a few questions regarding the work.

We appreciate the Reviewer's questions, which have allowed us to clarify aspects of our work.

In page 7, parameter calibration should be separately done on the samples with WGD.

*In Results Section 2 (**Fig. 2**), we infer the selection rates for non-WGD samples in each cancer type. In Results Section 4 (**Fig. 4**), we then infer the WGD probability, and WGD-aneuploidy rate (defined as the increase in missegregation probabilities in WGD cells, compared to non-WGD cells).*

We found that this strategy is more practical than calibrating parameters from scratch for WGD samples. This can be illustrated by the cancer evolution with WGD below:

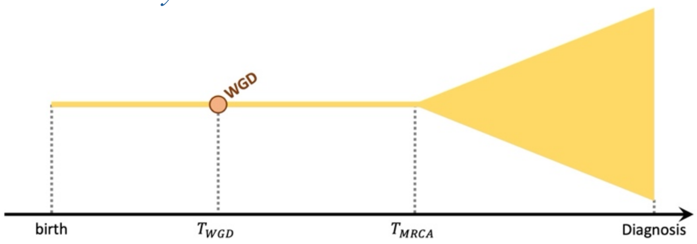

If we calibrate parameters from scratch for these samples, the parameters include:

- *44 chromosome-arm selection rates in non-WGD cells (from birth to* T_{WGD}*)*
- *44 chromosome-arm selection rates in WGD cells (from* T_{WGD} *to diagnosis)*
- *Probability of WGD*
- *Probabilities of missegregation in non-WGD cells*
- *Probabilities of missegregation in WGD cells*

In short, there are twice as many parameters to infer, compared to non-WGD samples. In our prepatory studies, CINner parameters become non-identifiable, and we are unable to extract biologically relevant information.

However, we note that similar to Section 2, the inference in Section 4 is performed for each cancer type separately. The data used for fitting in this Section consists of WGD samples, as opposed to only non-WGD samples in Section 2.

In page 19, the authors mentioned that CINner can model rare cancers which are limited by small sample size. But to infer the parameters, does CINner need a large sample size? How can it identify the driver gene given the small sample size?

Identifying driver genes in a cancer type requires studies which have identified recurrent CNAs or SNVs independently observed across many patients, the sensitivity of which depend on large cohort sizes. Therefore, driver gene identification for rare cancers is challenging, given the small sample sizes.

*CINner is not intended to be a method for finding driver genes. Instead, it finds the selection parameters for each chromosome arm, which reflect the combined effect of oncogenes and TSGs on the arm (see comparison of CINner-inferred selection parameters for TCGA pan-cancer (n = 8207), and the Charm scores for the same arms, **Fig. 2l**). The focus on whole arms instead of individual genes allows the inference to be performed with much smaller sample sizes (see **Supplementary Table 1**, which lists PCAWG sample sizes for cancer-specific inference).*

We have added these sentences to clarify our claim: "For these cancer types, we can instead infer the chromosome arm selection parameters with significantly fewer samples (Fig. 2b-d, Supplementary Fig. 1-17). Each arm's selection parameter can then serve as an indicator for the combined impact of driver genes located on it." (lines 626-629)

Fig. 2g is not so clear to see the correlation between WGD proportion and the mean selection rate of the LOSS arm. A dot plot might be better to show the correlation.

*We changed **Fig. 2h-j** (previously Fig. 2g-i) to scatter plots, which demonstrate the correlation between the WGD proportion and each cancer-type-specific statistic inferred from CINner.*

It seems that the comparison between the fitted model and the data such as Fig. 2a and other figures used the data being compared to fit the model. This is not fair because it is expected that the model will fit the data if the data is used to come up with the model. Moreover, there is very limited comparisons with other methods. It is mostly just the comparison between the model and the data, whereas the model seems come from the data itself. The cross validation is needed in this case.

*We include **Fig. 2b-g** (previously **Fig. 2a-f**) only to confirm that the CINner simulations using inferred parameters are consistent with the data that was used for the inference. This is because a different model, e.g. one with selection rates derived from pan-cancer data (doi:10.7554/eLife.69799, doi:10.1371/journal.pcbi.1006447, doi:10.1016/j.celrep.2015.06.065), would not necessarily fit tissue-specific data well.*

*We agree with the Reviewer that these results only show that CINner can fit the input data well, and do not indicate whether the inferred parameters are biologically relevant. However, we are not aware of any other algorithm that can fit the selection parameters for distinct cancer types (see **Supplementary Table 2**).*

*We note that although we cannot compare CINner's performance against previous algorithms, we validate our results by testing its predictions for tissue-specific proportion of WGD. Because the inference in **Fig. 2b-g** uses only non-WGD samples, it is blind to the WGD prevalence. Therefore, that the selection parameters can predict WGD proportions (**Fig. 2h-j**) serves as validation of our results, and implies the biological relevance of our inferred selection parameters. This is further demonstrated when the selection parameters inferred using only gain/loss frequencies in TCGA pan-cancer data show significant correlation with arm-specific gene imbalances (**Fig. 2l**).*

The paper compared with only one method, which is citation 22. It shows that it is consistent with the score proposed by citation 22. But is it better than citation 22?

We note that CINner and the Charm scores (citation 22) employ significantly different methodologies.

The Charm scores result from a study across the TCGA pan-cancer cohort ($n = 8207$) that quantifies the total effect of oncogenes and TSGs on each chromosome arm, with or without essential genes. The authors then showed that these scores can predict the frequencies of gains and losses of these arms across all TCGA samples.

*Meanwhile, with CINner we fit the gain/loss frequencies in a data cohort, to find the selection parameters for each chromosome arm. That this approach, when applied to TCGA data, results in selection parameters strongly correlated with the Charm scores (**Fig. 2l**) indicate that our approach can measure the total gene imbalance without knowledge of individual driver genes.*

*We also note that the Charm approach, like other studies, require large sample sizes to resolve the role and potency of a given gene. CINner, on the other hand, can analyze data cohorts with much fewer samples, which is the case for distinct cancer types in PCAWG (see **Supplementary Table 1**). However, CINner cannot predict the role of individual driver genes. Therefore, CINner is a complementary approach to sequencing studies of large cohorts toward understanding cancer evolution.*

"Importantly, the selection parameter fitting routine only requires a small cohort of cancer samples". How many cancer samples are needed for the parameter fitting for each cancer type?

*We included **Supplementary Table 1**, which lists the number of non-WGD samples used for inferring the selection parameters in each cancer type. We note that most cancer types have relatively few samples, e.g. 8/17 cancer types have $n \leq 20$.*

Why in 3b the average ploidy never goes up to 4? Is it limited by the cancer types the authors analyzed?

*The parameter study in **Fig. 3a-d** focuses on analyzing how extreme selection rates impact sample statistics. This study is relevant, as some cancer types contain many CN losses (e.g., Kidney-ChRCC; **Supplementary Figure 7**), while others contain many CN gains (e.g., Stomach-AdenoCA; **Supplementary Figure 15**).*

For this study to be consistent, we base it instead on the parameters inferred for the TCGA pan-cancer dataset. We then increase the selection rates for either LOSS arms or GAIN arms, then study the changes in statistics observed in hypothetical samples, assuming they begin as diploid cell populations. Because we do not include whole-genome duplication, the observed CNAs are limited to missegregations, therefore the average ploidy is constrained between 1 and 3.

*We note that whole-genome duplication is studied in the next Section (**Fig. 4**).*

”In previous sections, we estimated cancer type-specific selection parameters and missegregation probabilities in diploid cell populations,” When the ploidy goes up to 3, it is no longer diploid. However, from the figure 3b, the ploidy does go up to 3. I wonder if it is authors ’wrong definition of diploid, or no cell’s ploidy goes beyond 2.

*In the original manuscript, we meant that the parameter inference (**Fig. 2**) was limited to samples without whole-genome duplication (WGD), and likewise the parameter studies (**Fig. 3**) do not include the effect of WGD, which is studied in **Fig. 4b-c**. In particular, in the parameter studies we expand the selection rates to extremes (with very high selection rates for GAIN arms and/or LOSS arms). The Reviewer is correct that in those cases, the (synthetic) samples would no longer be diploid.*

We have therefore changed the sentence to “In previous sections, we estimated cancer type-specific selection parameters and missegregation probabilities in cell populations without whole-genome duplication (WGD) (Fig. 2).” (lines 429-430)

The inference and comparison with PCAWG cohort in Fig. 4d is interesting. I wonder how consistent is counted as satisfactory. For example, Liver-HCC and Cervix-SCC are both not so consistent as other cancer types. Since the authors didn’t compare with any other method, it is hard to give a qualitative conclusion.

*The WGD proportion and WGD FGA difference for Liver-HCC and Cervis-SCC simulated from CINner differ slightly more from PCAWG observations, compared to other cancer types. However, they are well within the PCAWG confidence intervals (dotted bars with circles, **Fig. 4d**). This is due to small sample sizes across PCAWG cancer types (**Supplementary Table 1**).*

*A direct comparison with other methods requires that those algorithms support the simulation of WGD and chromosome and chromosome-arm missegregations, and the inference of arm selection parameters. We are not aware of any other such method (**Supplementary Table 2**). The most similar algorithm to CINner in capabilities is CINsim. However, CINsim’s inference method was designed for single-cell data from mouse T-cell lymphomas and human colon cancer organoids (doi:10.1101/2023.02.14.528596), and does not currently support bulk DNA data.*

*We note that despite small sample sizes, the trend in the PCAWG data is clear: an increase in WGD proportion is associated with a decrease in WGD FGA difference across different cancer types (**Fig. 4d**). This trend is recreated in CINner simulations, produced with parameters that were inferred independently for each cancer type (**Fig. 4d**). This results in a clear negative correlation between WGD probability and WGD-aneuploidy rate (**Fig. 4e**). Therefore, we argue that the inference is consistent enough to support our findings.*
