# Supplementary material for: CINner: Modeling and simulation of chromosomal instability in cancer at single-cell resolution
Source: PLoS Comput Biol. 2025 Apr 3;21(4):e1012902. doi: 10.1371/journal.pcbi.1012902 (PMC11990800; doi:10.1371/journal.pcbi.1012902)
Supplement: Attachment — Submitted filename: Dinh_et_al_review.docx [file pcbi.1012902.s028.docx]

Dinh et al. present an ambitious model of chromosomal instability and cancer evolution with an extensive, clear, and detailed supplementary methods section. This work is an exciting approach allowing the incorporation of multiple of mechanisms of genome alteration in simulations and will likely be a useful addition to the literature. However, the manuscript (and online vignettes) require much significant work to improve their clarity before publication to ensure the method is used by others. It would be a real loss if this work is not communicated clearly. Firstly, simple steps to aid the reviewers such as including line numbers should be undertaken as well as ensuring text in figures is large enough to be read when printed, e.g. in Figures 3 and 4. Furthermore, significant changes including additional schematics and major improvements to existing figures should be made.

Concerns

1) The authors summarize the literature and contrast their approach with previous models. A main or supplementary table/figure detailing the differences in capabilities between the various previous models and CINer would be a useful addition. This table could also include potentially desirable features of copy number simulations that neither CINer nor other algorithms demonstrate – e.g. allele-specific copy number aberration simulation.
2) Figure 1 could be much improved and/or split into multiple main and supplementary figures. Given the computational audience of the journal, particular care should be taken to clearly demonstrate SNVs and the five distinct CNA mechanisms incorporated by CINer. I would suggest individual simple schematics featuring cartoon chromosomes for each followed by a by visualisations of CINer’s internal representation (current Figure 1b). Care should also be taken regarding the description of each mechanism. The legend for 1b states “Chromosome arm missegregation misplaces a random chromosome arm”, which does not adequately communicate the separate effects on the two daughter cells.

Figure 1c is also difficult to interpret with unreadable text and could be much improved. One issue is that the selection model is for chromosome arms, yet the reader is left to identify that arms are represented in the model only by the differences in selection indicated below each heatmap representation of chromosomes 2 and 3.

The legend for Figure 1e is inadequate. What do the vertical dashed arrows indicate. In addition, could the authors describe the various outputs of the method in detail in the supplementary or vignettes?

3) Many design choices are justified by “efficiency” – what are the computational demands of simulating the growth of tumor with this algorithm? What parameter
4) The parameters used by the model and parameter estimation program referenced on page 7 are insufficiently explained in the main text and figures. Schematic figure(s) briefly describing these as well as the analyses and their motivation performed in Figure 2 should be included to aid reader comprehension.
5) The PCAWG and TCGA acronyms are introduced without explanation. In addition, the numbers of cases analyzed should be made clear in supplementary tables and figure legends. The authors could also consider including the datasets and number of samples analyzed in the abstract.
6) The authors should make it clear that tissue and tumor-type specific patterns of aneuploidy are well known and described in the literature with appropriate references. In addition, care should be taken over language. For instance, the sentence “Cancer cells that have amplified GAIN arms are more selective” should be rewritten.
7) Given that the CINer is estimating selection parameters from individual cancer types it would be interesting to see a comparison between the CHARM scores that are estimated from pan-cancer SNV data and their own cancer-type specific scores. For example, for cancer types for which there are sufficient tumors in both PCAWG and the TCGA, could selection parameters be inferred from one dataset and examined on the other as well as being investigated with CHARM scores? One might expect that the cancer-type specific scores inferred by CINer would outperform the pan-cancer CHARM scores.
8) Could the authors clarify their meaning when they say “For instance, the genomes of WGD squamous cell lung carcinoma are significantly altered, but not at a substantially higher level than non-WGD tumours”. How is the word significantly being used here? When examining figure 4a the FGA in the WGD samples looks very different to the non-WGD samples, could the authors comment?
9) On page 12 a sample size is specified at 1000 cells, this seems low – what are the sample sizes used for all other simulations.
10) The authors repeatedly mention the limitations of bulk sequencing data and the possibility of applying CINer to single cell data. I note that the some of authors have previously published papers utilizing single cell datasets. Could the authors consider including a case study of single cell data that could be examined alongside a bulk data from the same cancer type? I understand that this may be beyond the scope of this paper and that the authors may not have bandwidth for such further analysis.
11) In the supplementary page 8, section 1.5.2, $\lambda_d \in (0,\infty)$ (para 1):

Presumably $\lambda_d$ is different for each driver gene? Or not? The sentence is ambiguous.

The next equations only seem to make sense if $\lambda_d \ge 1$.

The definitions of $\lambda_{d-WT}$ and $\lambda_{d_MUT}$ seem unmotivated and are unexplained. Would it not be cleaner to have a single style of parametrisation for both, for example the ONCs might have $\lambda_{d-WT}=1$, $\lambda_{d-MUT} = \lambda_d$, if $\lambda_d$ needs to be predefined as greater than 1 for some reason?

Equation (3) on the following page, defining a cell's fitness, seems not to include the non-linear effect on fitness of total loss of both viable alleles of a TSG. Is this intentional?  For example, if one arm containing an ONC/TSG is lost, and the corresponding on the remaining arm is mutated, is the combined model's estimate of fitness realistic? Are the nonlinear fitness effects that occur when LOH exposes a single mutated copy of a gene modelled adequately here?

Minor points:

The online Readme has a spelling error “Direct Library Prep**e**ration+ (DLP+).”
